# Modular RNA:DNA Nanostructures Enable Nanopore Profiling of rRNA Processing and rRNA Variants

**DOI:** 10.1021/acsnano.6c01132

**Published:** 2026-05-13

**Authors:** Filip Boskovic, Sarah E. Sandler, Simon Brauburger, Yuan Shui, Bhavik Kumar, Joana Pereira Dias, Plamena Naydenova, Jinbo Zhu, Stephen Baker, Ulrich F. Keyser

**Affiliations:** 1 Cavendish Laboratory, 2152 University of Cambridge, Cambridge CB3 0HE, U.K.; 2 University of Cambridge School of Clinical Medicine, Cambridge Biomedical Campus, Hills Road, Cambridge CB2 0SP, U.K.; 3 Department of Medicine, University of Cambridge School of Clinical Medicine, Cambridge Biomedical Campus, Hills Road, Cambridge CB2 0SP, U.K.; 4 A*STAR Infectious Diseases Laboratories (A*STAR IDL), Agency for Science, Technology and Research (A*STAR), Singapore 138648, Singapore

**Keywords:** solid-state nanopores, RNA:DNA nanostructures, rRNA processing, bacterial identification, *Salmonella* serotyping, single-molecule detection

## Abstract

Ribosomal RNAs (rRNAs) serve as species-defining markers and undergo processing steps, such as excision of intervening sequences (IVSs). Direct analysis of native rRNAs is hampered by enzyme-induced biases and by the high conservation of rRNA sequences, which complicate discrimination of closely related variants. Here, we present modular RNA:DNA nanostructures that enable direct identification of native rRNAs and their variants. The approach employs rationally designed RNA:DNA duplexes, named RNA identifiers (IDs), assembled onto native rRNAs via short complementary oligonucleotides bearing programmable coding motifs. We demonstrate that native bacterial 16S rRNAs can be directly converted to RNA IDs and detected with solid-state nanopores. Having established a direct rRNA readout, we next show that biologically encoded rRNA processing states, including serovar-specific 23S rRNA fragmentation patterns arising from IVS excision, are resolved using RNA IDs. Finally, to extend discrimination beyond processing-level differences, we incorporate catalytically inactive Cas9 ribonucleoprotein complexes to enable single-nucleotide discrimination of rRNA variants. Our modular RNA ID nanopore system facilitates the study of rRNA processing and rRNA diversity.

## Graphical abstract

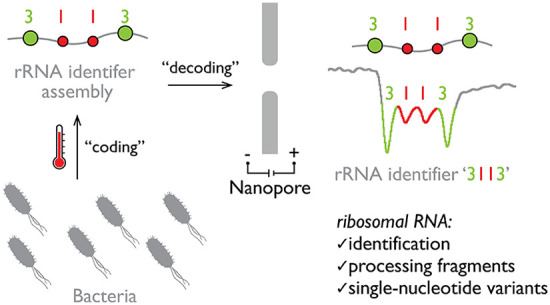

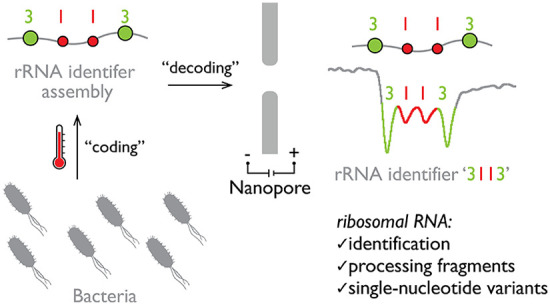

## Introduction

Ribosomal RNA (rRNA) is a defining molecular feature of bacteria, functioning both as a core structural component of the ribosome and as an informative marker of species-level identity.^1–6^ Bacterial rRNA sequence, processing patterns, and intervening sequence (IVS) presence reflect evolutionary divergence.^7–11^ Because rRNA is produced at high copy numbers during active growth,^12^ it provides a rich and abundant target for identifying bacterial serovars, rRNA maturation products, and lineage-specific sequence variation. These properties make rRNA an attractive molecule for probing species rRNA diversity and biology directly at the RNA level, rather than through indirect inference from rRNA gene sequences.^13,14^

Although rRNA encodes rich information, experimentally accessing this diversity remains challenging. Many approaches infer rRNA characteristics indirectly from gene sequences or rely on enzymatic amplification steps, including quantitative polymerase chain reaction (PCR), Illumina-based sequencing approaches, and RNA nanopore sequencing, which can limit access to native rRNA molecules and their processing intermediates. Enzyme-introduced errors can lead to substantial loss of apparent rRNA diversity, misassigning species, and introducing quantification errors.^3,4,15^ These workflows can mask features such as IVS-containing products, complicate quantitative interpretation of full-length rRNAs, and introduce sequence- or primer-dependent biases.^16^ In addition, the large size and complex secondary structure of rRNA pose practical challenges for direct analysis, even with promising long-read sequencing strategies that do not yet provide sufficient fidelity to detect such diversity of native full-length RNA.^17–19^ Consequently, complementary approaches that enable direct interrogation of native rRNAs while preserving their processing information would be valuable.

Herein, we introduce modular RNA:DNA nanostructures that enable direct analysis of full-length bacterial rRNAs without enzymes. Short complementary oligonucleotides bearing programmable structural elements are used to reshape native rRNAs into defined RNA identifiers (IDs), which generate characteristic current signatures upon translocation through solid-state nanopores. The ionic current signature of a nanopore translocation reflects the charge, size, and shape of the molecule traversing the pore. The workflow integrates rRNA extraction and RNA ID assembly into a single preparation step while limiting thermal degradation and background RNA. Using this approach, we resolve IVS-containing 23S rRNA processing intermediates from *Salmonella enterica* subsp. *enterica* (*Salmonella)* serovars and detect both rRNA and strain-specific mRNA from *Acinetobacter baumannii*. High specificity is demonstrated by the lack of detectable RNA ID nanopore events when total *A. baumannii* RNA is challenged with hundreds of rRNA oligonucleotides designed for other rRNA species. Incorporation of dCas9–guide RNA complexes further extends the platform to single-nucleotide–resolved discrimination at targeted rRNA variant sites. Together, our approach establishes a generalizable strategy for high-specificity, single-molecule analysis of rRNA IVS processing and sequence heterogeneity.

## Results

### Bacterial Full-Length rRNA Identification with RNA Identifiers

Total cellular RNA contains folded rRNAs, including 16S and 23S rRNAs, embedded within a complex mixture of native RNA species (Figure 1a). To convert a selected target into a nanopore-readable construct, we added pools of short complementary DNA oligonucleotides (typically ∼38 nt; exact sequences are listed in the corresponding tables) and applied heating in the presence of excess oligonucleotides. Because these DNA strands fully complement the target rRNA, they reshape the folded native RNA into a linear RNA:DNA hybrid duplex, which we term an RNA identifier (RNA ID) (Figure 1a).^20^ Selected oligonucleotides within this pool were designed to contain an additional overhang that hybridized a 3′-biotinylated imaging strand. Each selected oligonucleotide hybridizes to a single defined site on the target RNA, producing a position-specific signal. Binding of monovalent streptavidin to one such biotinylated imaging strand generated a single structural label, defined as structural (pseudo)­color “1”. When two adjacent oligonucleotides carried this same motif, they generated structural color “2”, and when three adjacent oligonucleotides carried it, they generated structural color “3”. These structural labels produced larger ionic current blockades during nanopore translocation and thereby encoded the RNA ID. Thus, full complementarity first converts an otherwise folded and nanopore-indistinguishable native rRNA into a defined linear RNA:DNA hybrid duplex, and structural labeling then adds a second layer of specificity by producing a characteristic nanopore current signature (Figure 1).

**Figure 1:**
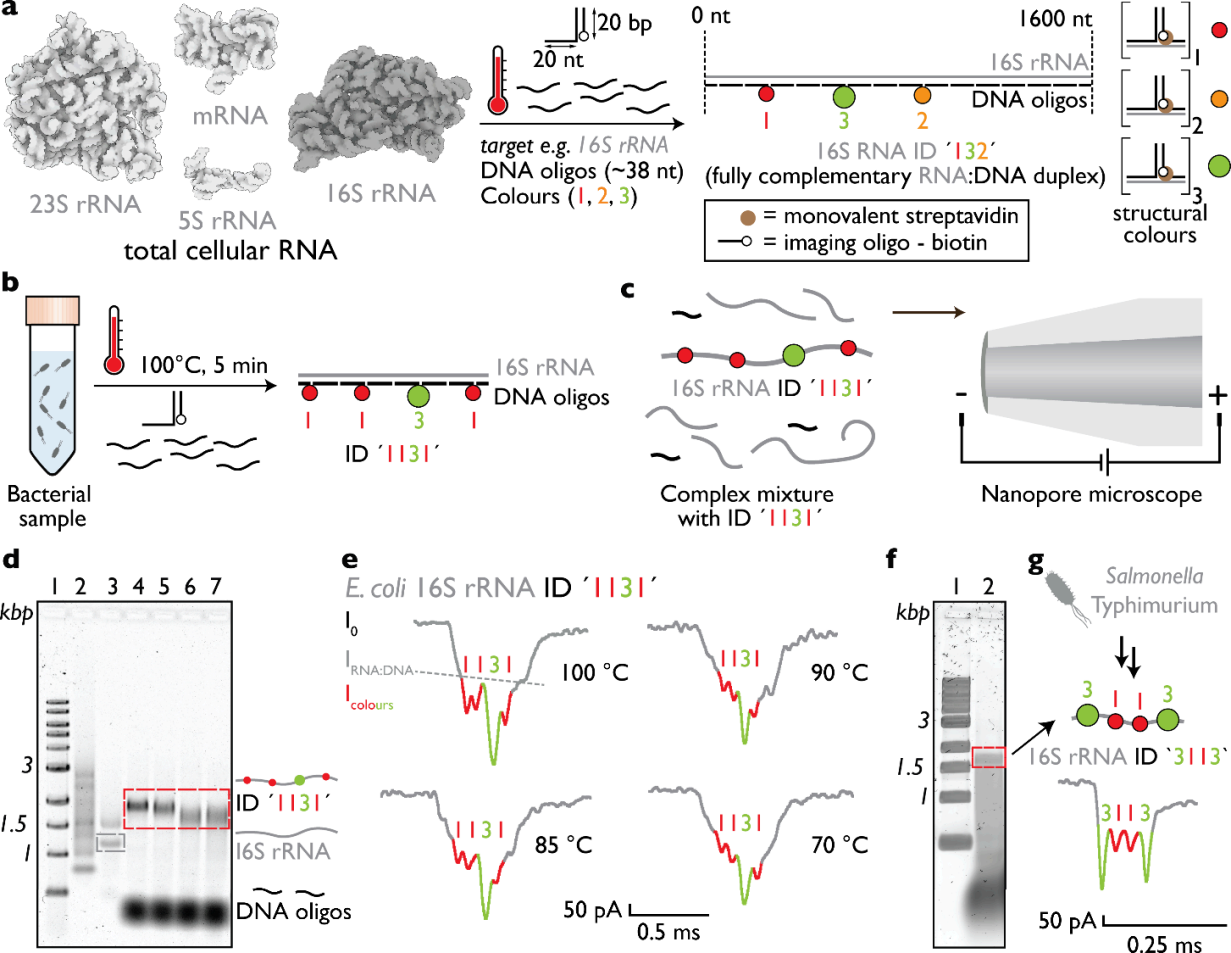
Simultaneous isolation, assembly, and nanopore detection of full-length rRNA IDs. (a) RNA ID assembly from total cellular RNA. Total cellular RNA contains folded native rRNAs, including 16S and 23S rRNAs. Upon addition of short complementary DNA oligonucleotides (∼38 nt), the target rRNA is reshaped into a fully complementary linear RNA:DNA hybrid duplex (RNA ID). Selected oligonucleotides bear overhangs that hybridize 3′-biotinylated strands, which bind monovalent streptavidin to generate structural pseudocolors “1”, “2”, and “3”. In this way, folded target rRNA, such as 16S rRNA, is converted into a defined RNA ID, exemplified here by 16S rRNA ID “132”. (b) Heating an *E. coli* culture at 100 °C for 5 min in the presence of complementary DNA oligonucleotides enables concurrent rRNA release and assembly of RNA IDs. The 16S rRNA transcript is reshaped into a defined RNA:DNA hybrid duplex through oligonucleotide binding, and positioning of structural labels at defined sites generates the *E. coli*-specific 16S rRNA ID “1131”. (c) Engineered RNA IDs can be detected directly in complex lysates using nanopore microscopy. (d) Agarose gel electrophoresis (1% w/v) of *E. coli* total RNA before and after RNA ID assembly shows intact 23S and 16S rRNAs prior to treatment (lane 3), whereas samples heated at 70, 85, 90, or 100 °C (lanes 4–7) display selective enrichment of the assembled 16S rRNA ID “1131” accompanied by degradation of unpaired 23S rRNAs. Lane 1 corresponds to a 1 kbp DNA ladder and lane 2 to an ssRNA ladder. (e) Representative nanopore ionic current traces collected over 10 min measurements demonstrate detection of characteristic RNA ID signals for all assembly temperatures. In an RNA ID nanopore event, *I*_0_ denotes the open-pore ionic current, *I*_RNA:DNA_ the current level associated with translocation of the RNA:DNA duplex, and *I*_colors_ the additional downward current spikes generated by the structural labels. (f) Direct assembly of RNA IDs from *Salmonella*Typhimurium culture without prior RNA purification yields an approximately 1.6 kbp 16S rRNA ID detectable by agarose gel electrophoresis. (g) The generality of the approach is demonstrated by assembly and nanopore detection of the 16S rRNA ID “3113” from *S. Typhimurium* culture.

We hypothesized that RNA ID assembly with DNA oligonucleotides could occur during bacterial lysis. To test whether the elevated temperatures required for rapid rRNA release would degrade rRNA during assembly, *E. coli* cells were heated to 70, 85, 90, or 100 °C for 5 min in the presence of DNA oligonucleotides (Figure 1b; sequences listed in Table S1). In this proof-of-concept experiment, *E. coli* 16S rRNA was selected as the target. Our design incorporates both the “1” and “3” structural codes, which denote engineered structural motifs of distinct sizes.^20^ Assembly of these elements yields a unique RNA:DNA hybrid duplex, designated RNA ID “1131”. The “1131” code also corresponds to the characteristic shape of the nanopore current signal generated by this RNA ID (Figure 1c). This distinct current signature enables detection of 16S rRNA even in complex mixtures containing cellular biomacromolecules and debris such as other RNAs, DNA, lipids, and proteins.

We validated RNA ID assembly using both agarose gel electrophoresis and nanopore measurements by heating *E. coli* DH5α total RNA for 5 min at different temperatures (Figure 1d,e). Prior to treatment, total RNA (lane 3) exhibited two dominant bands corresponding to 23S and 16S rRNA, whereas 5S rRNA was not readily resolved under these gel conditions, consistent with its much smaller mass contribution^21^ (Figure 1d). After applying the RNA ID assembly protocol to generate 16S rRNA ID “1131” (lanes 4–7), the 23S rRNA band was no longer observed, indicating preferential degradation of unpaired rRNAs during the heating step. Successful RNA ID assembly was observed at all four temperatures tested (70, 85, 90, and 100 °C), as evidenced by the remaining band corresponding to the assembled 16S rRNA ID (lanes 4–7). These results indicate selective enrichment of the targeted 16S rRNA ID, highlighted by the red dashed box in Figure 1d.

We next assessed RNA ID assembly at the single-molecule level for all temperature conditions (Figure 1d; additional events shown in Figure S1). In all cases, the characteristic “1131” barcode was detected during a 10 min nanopore measurement. The nanopore results were consistent with gel electrophoresis observations.

Finally, we applied the heating-based assembly protocol directly to bacterial cultures.*S.* samples mixed with DNA oligonucleotides were heated at 100 °C to assemble 16S rRNA ID “1131” (Figure 1f). Agarose gel electrophoresis revealed a band corresponding to the assembled ≈1.6 kbp RNA ID (Figure 1f, lane 2). We further demonstrated the generality of this approach using *S. Typhimurium*, targeting its 16S rRNA to generate RNA ID “3113”. Single-molecule nanopore events corresponding to this RNA ID were detected within a 10 min measurement (Figure 1g; additional events shown in Figure S2). Together, these results show that RNA extraction, RNA ID assembly, and enrichment can be combined into a single step, enabling efficient identification of full-length bacterial rRNAs at the single-molecule level.

### Discriminating *A. baumannii* Variants Using rRNA and mRNA RNA IDs

We next applied this approach to *A. baumannii*, a bacterium known for extensive genomic and transcriptomic variation, including differences associated with strain-specific genetic determinants.^22,23^ One such feature is the presence of the glycosyltransferase gene *gtr100*, which encodes a capsule-associated modification and is present in specific lineages including KL49 strains.^22^ This system therefore provides a useful model to test whether the RNA ID strategy can resolve closely related bacterial strains by simultaneously targeting abundant rRNA and mRNA transcripts.

We designed RNA IDs targeting both the 16S rRNA and the *gtr100* mRNA of a KL49 strain, assembling them into RNA IDs “331” and “3113”, respectively (Figure 2a; sequences listed in Tables S2 and S3). Total RNA from two samples, *A. baumannii* lacking *gtr100* (control) and *A. baumannii* KL49 expressing *gtr100*, was analyzed using nanopore measurements (Figure 2b). In both samples, the 16S rRNA RNA ID was readily detected, consistent with the presence of conserved ribosomal transcripts (Figure 2c; additional events shown in Figure S3). In contrast, the RNA ID corresponding to the *gtr100* mRNA was detected exclusively in the KL49 sample, reflecting strain-specific transcript expression.

**Figure 2:**
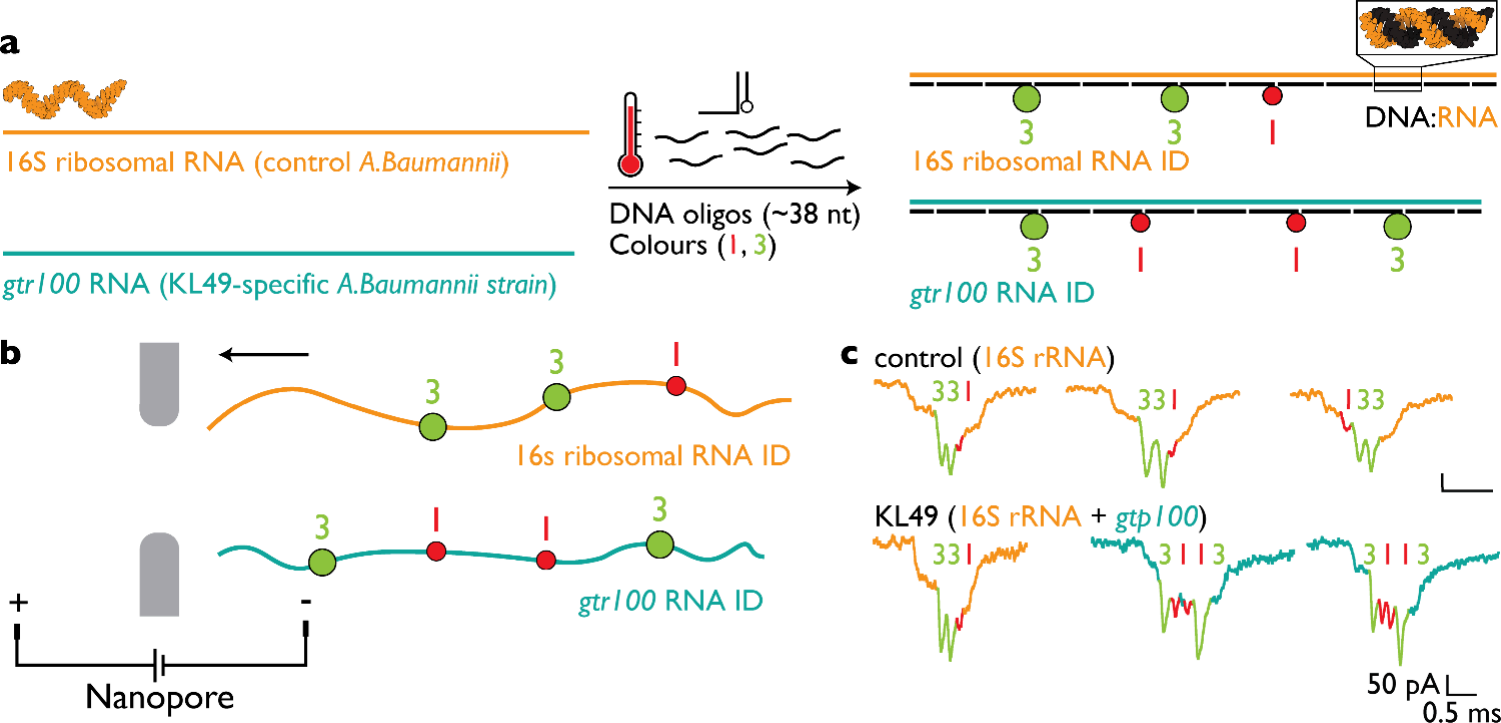
Parallel identification of rRNA and a strain-specific transcript in *Acinetobacter baumannii*. (a) RNA IDs were designed to target two RNA species: the 16S rRNA common to *A. baumannii* and the mRNA encoded by the strain-associated gene *gtr100*. Assembly of these targets yielded a 16S rRNA ID “331” and a *gtr100* mRNA ID “3113”, respectively. (b) Schematic representation of nanopore measurements used to analyze RNA IDs assembled from total RNA extracted from two *A. baumannii* strains. (c) Representative nanopore events demonstrate detection of the targeted RNA species. The 16S rRNA ID “331” was observed in RNA samples from both strains, whereas the *gtr100* mRNA ID “3113” was detected only in the KL49 strain, consistent with strain-specific transcript expression.

As expected, the *gtr100* mRNA was present at substantially lower abundance than 16S rRNA. Detection of the corresponding gtr100 RNA ID signals occurred at frequencies of approximately 10^–3^–10^–4^ per event. Together, these results demonstrate that the RNA ID nanopore approach can distinguish closely related *A. baumannii* variants by parallel analysis of abundant rRNA and mRNA targets.

### RNA IDs Enable Multiplexed Species Identification and Coculture Tracking

We evaluated whether RNA IDs could support identification of rRNA transcripts from multiple species or multiplexed tracking within a single experimental framework. Total RNA samples from four phylogenetically distinct sources, human, mouse, *E. coli*, and *S. Typhimurium*, were analyzed to test whether species-specific rRNAs could be selectively converted into RNA IDs (Figure S4). Eukaryotic 18S rRNA and prokaryotic 16S rRNA transcripts were targeted as species-defining RNA molecules (Figure S4a). Four RNA IDs were designed accordingly: “1131” for *E. coli* 16S rRNA, “1111” for human 18S rRNA, “3232” for mouse 18S rRNA, and “3113” for *S. Typhimurium* 16S rRNA (Figure S4b; sequences listed in Tables S1, S4, S5, and S6). Distinct nanopore current signatures corresponding to each RNA ID were observed, demonstrating that multiple rRNA targets can be identified in parallel (additional events shown in Figures S1 and S5–S7).

We further extended this approach to mixed nucleic acid samples containing bacterial and viral components to assess their ability to resolve multiple species simultaneously. RNA IDs were assembled to target *E. coli* 16S rRNA (ID “1131”), the single-stranded RNA genome of bacteriophage MS2 (ID “111”), and the single-stranded DNA genome of bacteriophage M13 (ID “111111”) using a mixture containing *E. coli* total RNA, MS2 RNA, and M13 DNA (Figure.S8; sequences listed in Tables S1, S7, S8, and S9). Nanopore measurements revealed distinct current signatures corresponding to each nucleic acid target, indicating successful parallel identification of bacterial rRNA, viral RNA, and viral DNA within a single sample (Figure S8).

To assess the specificity of RNA ID assembly, we challenged the system with a large excess of nontargeting oligonucleotides. A pool of more than 500 oligonucleotides designed to target human 18S rRNA, mouse 18S rRNA, *E. coli* 16S rRNA, and *Salmonella* 16S and 23S rRNAs was mixed with total RNA from *A. baumannii* (Figure S9a). RNA ID formation requires full complementarity of the added oligonucleotides to the target rRNA,^20,24^ and noncomplementary oligonucleotide libraries do not produce linearized RNA ID structures or the corresponding nanopore signatures (Figure S9). Agarose gel electrophoresis and extended nanopore measurements were then performed to detect any evidence of nonspecific RNA ID formation, including the *A. baumannii* 16S rRNA ID “331” (Figure S9b,c). Across more than 10 h of measurement time and thousands of nanopore events consistent with RNA or double-stranded DNA molecules, no RNA ID signatures were detected. These results indicate the high specificity of RNA ID assembly for the intended target sequences, which we attribute to the combined effects of selective RNA degradation during the heating and assembly step and the distinctive nanopore signals produced by correctly assembled RNA IDs.

### *Salmonella enterica* Serovar Identification by Fragmented 23S rRNA

Having established rRNA-based species identification, we next extended the RNA ID approach to analyze naturally fragmented rRNA. Several *Salmonella enterica* serovars contain an IVS within their 23S rRNA genes with differential combinatorics, which are excised by RNase III during rRNA processing, resulting in defined fragmentation patterns rather than a single full-length transcript^8^ (Figure 3a). Serovars lacking IVSs produce intact 23S rRNA, whereas serovars containing IVS1 or IVS2 generate characteristic sets of rRNA fragments following IVS excision.

**Figure 3:**
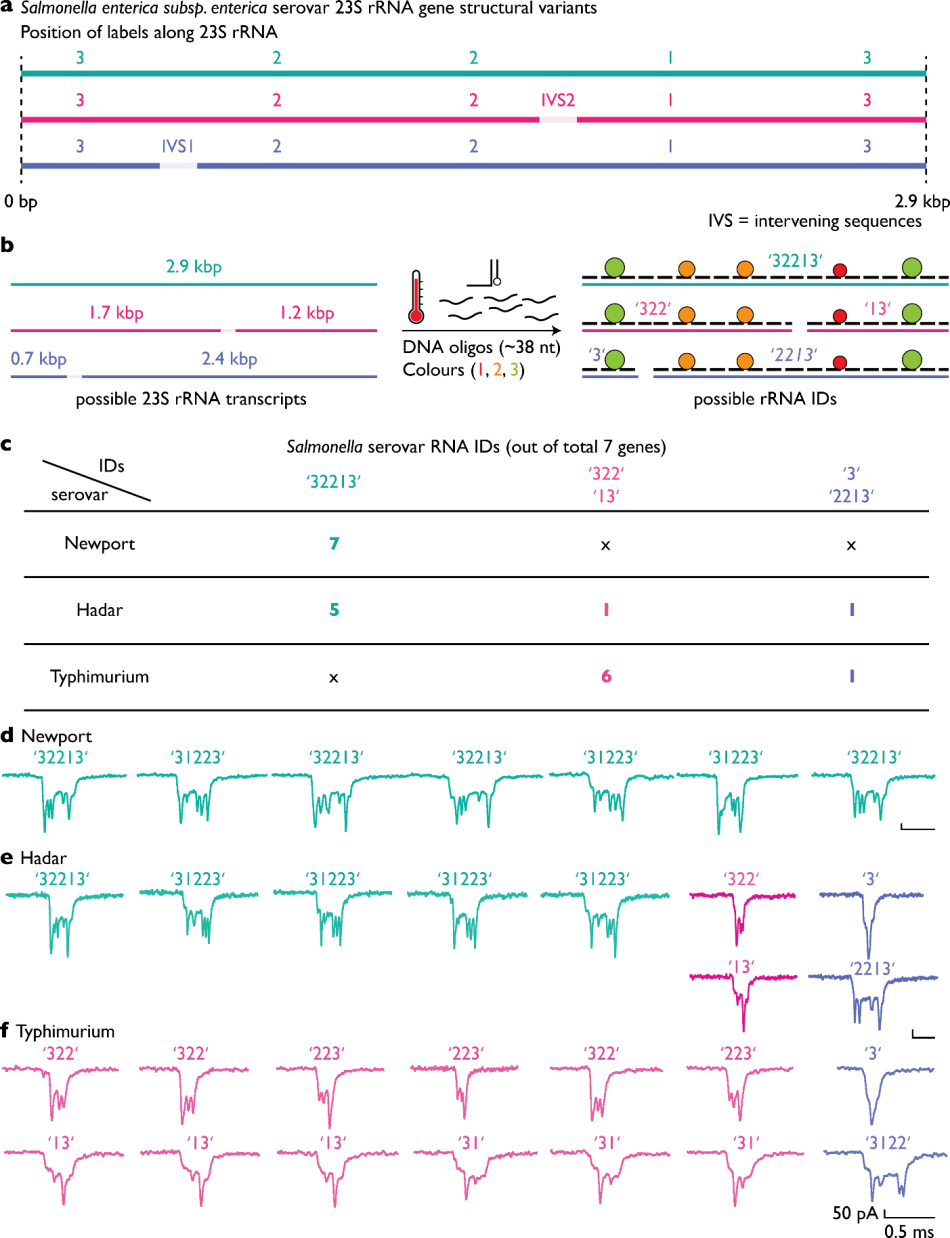
Resolving 23S rRNA processing products from IVS combinatorics using RNA IDs. (a) Schematic overview of 23S rRNA processing outcomes arising from different 23S rRNA gene variants. A full-length 2.9 kb 23S rRNA lacking intervening sequences yields the intact RNA ID “32213”, whereas variants containing IVS1 or IVS2 undergo RNase III–mediated excision, producing fragment pairs “322” and “13”, or “3” and “2213”, respectively. (b) Assembly of RNA IDs using a single oligonucleotide set generates distinct RNA IDs corresponding to each full-length or processed 23S rRNA. (c) Distribution of 23S rRNA gene variants across seven gene copies for the analyzed *Salmonella* serovars, expressed as the number of genes out of seven producing each processing rRNA product. *Salmonella* Newport contains exclusively full-length 23S rRNA genes and yields only RNA ID “32213” (7 of 7), *Salmonella* Hadar contains a mixture of all three variants with predominance of the full-length form (5 of 7), and *Salmonella* Typhimurium contains IVSs in all gene copies and therefore lacks the full-length RNA ID “32213” (0 of 7). (d) Representative nanopore events corresponding to 23S rRNA RNA IDs from *Salmonella* Newport. (e) Representative nanopore events corresponding to mixed full-length and fragmented 23S rRNA RNA IDs from *Salmonella* Hadar. (f) Representative nanopore events corresponding exclusively to fragmented 23S rRNA RNA IDs from *Salmonella* Typhimurium. RNA IDs translocate through nanopores bidirectionally and may therefore be detected in either orientation.

To capture 23S rRNA processing states, we designed an RNA ID architecture that assigns distinct structural codes to full-length and fragmented transcripts (Figure 3b). In this scheme, the full-length 23S rRNA is encoded as ID “32213”. Transcripts containing only IVS2 or IVS1 yield fragment IDs “322” and “13”, or “3” and “2213”, respectively. The expected lengths of all possible ≈2.9 kb 23S rRNA-derived transcripts corresponding to these IDs are summarized in Figure 3b.

We tested this strategy using total RNA from three *Salmonella* serovars: Typhimurium, Newport, and Hadar. Each serovar contains seven 23S rRNA gene copies, which differ in their IVS content. In the Typhimurium sample, six genes contain IVS2, and one gene contains IVS1. Using a single oligonucleotide mixture, this composition predicts the presence of fragment IDs “322”, “13”, “3”, and “2213”, with no full-length 23S rRNA ID expected (Figure 3c). In contrast, Newport lacks IVSs in all 23S rRNA genes and is therefore expected to predominantly yield the full-length 23S rRNA ID “32213”.

Following RNA ID assembly, all three samples were analyzed using nanopore measurements. Representative nanopore events for Newport, Hadar, and Typhimurium, are shown in Figure 3d, Figure 3e, and Figure 3f, respectively. Because RNA IDs can translocate through the nanopore from either end, individual events may appear in either orientation, for example, “32213” or “31223” for the full-length 23S rRNA ID.

Consistent with the predicted rRNA processing patterns inferred from their respective genomes, nanopore measurements revealed predominantly full-length 23S rRNA IDs “32213” for Newport (Figure 3d), a mixture of full-length and fragmented IDs “32213”, “3”, “322”, “13”, and “2213” for Hadar (Figure 3e), and exclusively fragmented IDs “322”, “13”, “2213”, and “3” for Typhimurium (Figure 3f). These results demonstrate that serovar-specific 23S rRNA processing patterns can be resolved at the single-molecule level using RNA IDs and nanopore readouts. To further extend the resolution of our approach, we next sought to determine whether sequence-level variation within rRNA could also be targeted, motivating the incorporation of dCas9 ribonucleoprotein (RNP) complexes to enable single-nucleotide–specific rRNA discrimination.^25^

### Catalytically Inactive Cas9 Enables Single-Nucleotide–Resolved rRNA Discrimination

To extend RNA ID–based discrimination beyond rRNA processing patterns and into rRNA-level variation, we next incorporated catalytically inactive Cas9 (dCas9) RNP complexes to target single-nucleotide differences within rRNA. dCas9 forms a programmable RNP complex with a guide RNA (gRNA) that binds complementary RNA^26^ in an RNA:DNA hybrid context, and under appropriate design constraints can exhibit single-nucleotide specificity. Here, we leverage this programmability to resolve closely related *S. enterica* serovars based on single-base differences in their rRNA sequences (Figure 4).

**Figure 4:**
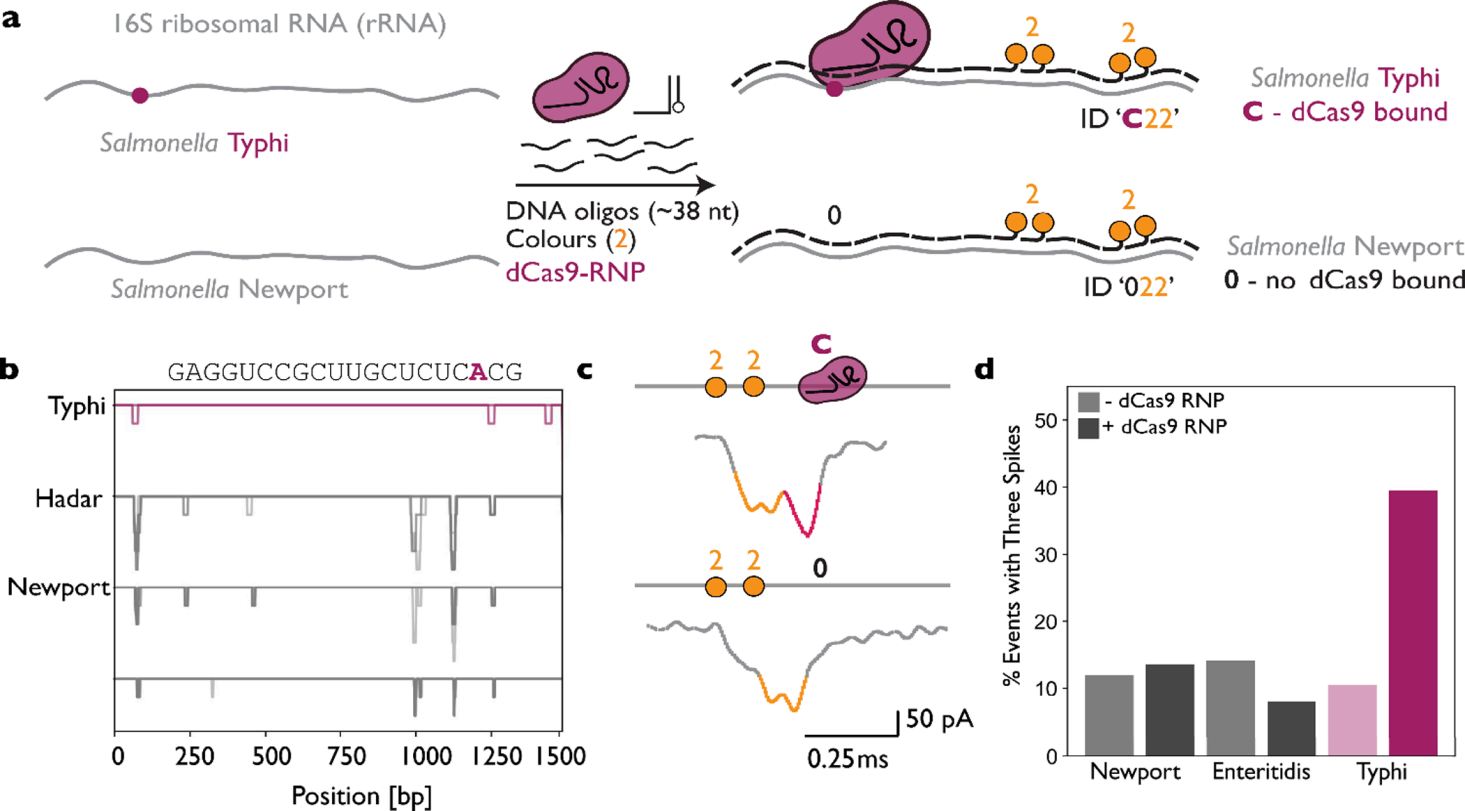
Single-nucleotide discrimination of Salmonella enterica rRNA variants using inactive Cas9 (dCas9) binding to RNA IDs. (a) Schematic representation of RNA ID assembly for two closely related 16S rRNA sequences from *Salmonella* serovar Newport and Typhi that differ by a single nucleotide. The rRNA transcripts serve as scaffolds for complementary DNA oligonucleotides, forming double-stranded RNA:DNA IDs containing defined structural elements corresponding to the bit “2”, each associated with two streptavidin molecules bound to biotinylated oligonucleotides. Sequence-specific binding of a dCas9 RNP complex to the Typhi rRNA ID introduces an additional structural element denoted as “C”, yielding the barcode “22C”, whereas the Newport rRNA ID lacks dCas9 binding and yields the barcode “022”. (b) Identification of single-nucleotide variant positions by sequence alignment of 16S rRNA from *Salmonella* serovar Typhi, Hadar, Newport, and Enteritidis, highlighting candidate target sites for dCas9 recognition. (c) Representative nanopore events corresponding to RNA IDs “22C” and “022”, showing an increased occurrence of three-spike events associated with dCas9-bound RNA IDs in samples containing Typhi. Event counts correspond to measurements performed on samples without Typhi (*N* = 32) and with Typhi (*N* = 30). (d) Quantification of the fraction of nanopore events containing three peaks before and after addition of dCas9 for Newport, Enteritidis, and Typhi samples. For Newport and Enteritidis, the fraction of three-peak events remains similar with or without dCas9, whereas Typhi shows an increase from approximately 10% without dCas9 (*N* = 45) to approximately 35% with dCas9 (*N* = 33).

In order to use this tool, careful design of the gRNA probes is essential. The binding of dCas9 to RNA:DNA hybrids is constrained by the presence of a protospacer-adjacent motif (PAM) on the DNA strand and by mismatch sensitivity within the gRNA sequence. Previous studies have shown that mismatches proximal to the PAM, particularly within the PAM-proximal “seed” region, most strongly impair Cas9 binding.^27^ Variants positioned distally from the PAM often have reduced mismatch sensitivity. Across *Salmonella* serovar, multiple candidate sites meeting these criteria were identified by 16S rRNA sequence alignment (Figure 4b and Figure S10). Candidate dCas9 RNPs were screened for single-nucleotide specificity using a previously established assay for DNA targeting,^25^ and representative measurements are shown in Figure S11 (sequences listed in Tables S8, S10, and S11). From these candidates, the gRNA targeting a single-nucleotide variant present at the highest frequency among 16S rRNA gene copies was selected for further analysis.

The selected gRNA that was used for an initial demonstration allows us to differentiate between *Salmonella* Typhi and Newport, which differ by a single nucleotide at position 1258 of the 16S rRNA (Figure 4a,b). A dCas9 RNP was assembled with a gRNA complementary to the Typhi sequence at this position. Binding of the dCas9 RNP to the 16S rRNA RNA ID introduces an additional structural feature, which is encoded as the barcode “22C”, where “C” denotes the presence of bound dCas9 and each numeric element corresponds to streptavidin-associated structural features as described above. A unique dCas9 nanopore signature has been demonstrated for dsDNA,^25,28,29^ and, similarly, when dCas9 is bound to RNA:DNA, “C” is detectable. In contrast to Typhi, the Newport 16S rRNA contains a single-base substitution at the target site, preventing dCas9 binding and yielding the barcode “022”. Representative nanopore events corresponding to these two RNA ID configurations are shown in Figure 4c, demonstrating discrimination between the two rRNAs based on a single-nucleotide difference.

Using this optimized dCas9 RNP, we compared 16S rRNA RNA IDs assembled from *Salmonella* Typhi, Enteritidis, and Newport (assembly sequences for RNA ID “022” listed in Table S12). Nanopore measurements revealed a higher relative frequency of RNA ID events containing the “22C” barcode in the Typhi sample relative to the other serovar, which predominantly yielded “022” events lacking dCas9 binding (Figure 4d). Independent repeats of RNA ID assembly and nanopore measurement for Typhi produced consistent results (Figure S12).

Finally, we tested whether dCas9-mediated discrimination could be maintained in mixed serovar samples. Two mixtures were analyzed, one containing Enteritidis and Newport only, and a second containing Enteritidis, Newport, and Typhi (Figure S13a). Nanopore events were classified based on the presence (“22C”) or absence (“022”) of dCas9 binding, with representative events shown in Figure S13b. Consistent with expectations, “22C” events were observed only in mixtures containing Typhi, demonstrating that dCas9-enabled RNA IDs can resolve single-nucleotide rRNA variants even in the presence of closely related sequences.

## Discussion

By converting native rRNAs into programmable RNA IDs readable by solid-state nanopores, we demonstrate that abundant rRNA products are reshaped into distinct, information-rich signals without reverse transcription or amplification. Amplification-free detection with RNA:DNA nanostructures has been demonstrated previously, including detection at femtomolar concentrations, and the present work extends this approach to profiling native full-length bacterial rRNAs and their variants.^20,30^ By reshaping full-length rRNAs into RNA IDs with defined structural signatures, we enabled single-molecule rRNA detection, 23S rRNA processing state specific to *Salmonella* serovars, and 16S rRNA sequence variation specific to serovars with dCas9 RNP binding.

Solid-state nanopores have been widely applied to the analysis of DNA,^31–35^ viruses,^36–38^ proteins,^39–42^ and RNA,^20,39,43–46^ including studies of complex biological assemblies.^47,48^ Previous nanopore-based RNA measurements have typically relied on purified targets or have been limited in specificity when applied to heterogeneous samples.^44,46,47^ The RNA ID builds on these strengths by introducing programmable structural encoding,^20^ which enables selective barcoding of RNAs of interest and discrimination from background nucleic acids within complex mixtures. Direct nanopore analysis of native RNA is especially valuable for studying RNA variants and isoforms that differ in length or structure, features that are difficult to preserve through enzymatic workflows.^20,30,49,50^ At the same time, RNA:DNA duplexes translocate faster than DNA:DNA duplexes,^51^ highlighting the importance of continued development of velocity-control strategies to further expand nanopore spatial resolution.^52–55^

Ribosomal RNA processing is inherently complex and variable across species, in part because rRNAs are encoded by multiple gene copies that can undergo distinct processing pathways.^1,7,10,13^ As a result, a single cell can contain a diverse population of rRNA products arising from differential cleavage, excision of IVS, and regulated maturation steps. These processes are increasingly recognized as responsive to genetic context and cellular conditions,^13,56–59^ underscoring the importance of accessing full-length, native rRNA populations. The RNA ID approach provides a complementary route to studying 16S and 23S rRNA transcripts by facilitating direct readout of rRNA products without enzymatic modifications of the RNA itself. This is particularly relevant for transcripts that are highly structured or modified, which may be inefficiently reverse-transcribed or underrepresented in sequencing-based workflows unless specifically accommodated by experimental design.^60,61^

Sequence-level discrimination within rRNA remains challenging due to the high conservation of ribosomal sequences.^3,4,15,18,62^ Incorporation of dCas9 RNP complexes adds an additional, programmable recognition layer to the RNA ID framework, enabling single-nucleotide–resolved discrimination within otherwise similar rRNAs. dCas9-based targeting has previously been employed for specific RNA detection,^26,63,64^ and its application herein to RNA:DNA hybrid nanostructures demonstrates a promising route for introducing sequence selectivity without altering the underlying RNA ID architecture. Such an approach is particularly relevant for distinguishing closely related bacterial serovars, where single-nucleotide differences within rRNA can carry important biological meaning.^65^ However, the applicability of a dCas9-based approach is constrained by the requirement that the distinguishing nucleotides lie sufficiently close to a protospacer-adjacent motif (PAM) to enable selective dCas9 binding. Future extensions of the RNA ID framework could incorporate alternative programmable RNA or DNA binding proteins that do not rely on PAM recognition, potentially expanding the range of accessible sequence variants.

## Conclusions

While our study focuses on capabilities for rRNA detection, the abundance of rRNA and the ability to resolve species-, serovar-, and variant-level differences suggests potential relevance for future diagnostic applications. Translation to clinical settings will require careful consideration of sample handling, RNA stability, and quantitative interpretation in complex biological environments. Our work provides a route for converting native rRNA molecules into programmable nanostructures, paving the way for single-molecule studies of rRNA processing and rRNA heterogeneity.

## Methods

### *Salmonella enterica* and *Acinetobacter baumannii* Cultures and Total RNA Isolation

Bacteria were cultured overnight in LB, and 500 μL of overnight culture was treated with two volumes of RNAprotect reagent (Qiagen). Cultures were digested with 15 mg/mL lysozyme (Sigma) for 20 min, and RNA extraction was performed using the RNeasy Mini Kit (Qiagen) following the manufacturer’s guidelines. Total RNA was quantified, and quality was assessed using NanoDrop (Thermo Fisher). Total RNAs from other samples were obtained from Thermo Fisher. The scaffold RNA sequences were obtained from either reference rRNA nucleotide sequence from the NCBI nucleotide database or the NCBI annotated genome database under accession numbers J01859.1 (*E. coli*), NR_074910.1 (*S.* Typhimurium), 16S rRNA sequence extracted from the annotated reference genomes 108619 (*S.* Newport), 149539 (*S.* Enteritidis), 149385 (*S.* Hadar), 90370 (*S.* Typhi), and KT359616.1 (*A. baumannii*).

### RNA ID Design

RNA IDs were designed by reshaping target RNA molecules into linear RNA:DNA hybrid nanostructures by using pools of short complementary DNA oligonucleotides. Oligonucleotides were typically 20 to 38 nt long, balancing stable hybridization to the target RNA with low intrinsic secondary structure.^20,30,49^ Shorter oligonucleotides can bind too weakly and be outcompeted by intramolecular RNA folding, whereas longer oligonucleotides are more likely to form secondary structures or multivalent interactions. Terminal oligonucleotides were extended with 3 to 5 dT residues to reduce stacking between RNA IDs. Assembly was performed by mixing total cellular RNA with the corresponding oligonucleotide pool in molar excess, followed by brief heating and cooling as described below. Under these conditions, the target RNA was converted from its native folded conformation into a linear RNA:DNA hybrid duplex, which provides a defined scaffold for nanopore readout.

To encode identity information, selected oligonucleotides contained a target-complementary region, a short dT linker to reduce steric hindrance, and a 20 nt overhang that hybridized to a 3′-biotinylated imaging strand. Each of these selected oligonucleotides has only one hybridization site on the target RNA, yielding a position-defined signal. Binding of monovalent streptavidin to this strand created a structural label whose steric volume generated an additional current blockade during nanopore translocation. A single such unit defined structural color “1”, while adjacent placement of two or three units generated structural colors “2” and “3”, respectively. In this study, four structural sites were typically used for 16S rRNA and five for 23S rRNA, providing sufficient spacing and information content for robust identification of full-length targets and selected fragments. Only fully complementary oligonucleotide sets produced the expected RNA IDs. Control experiments with nontarget oligonucleotide libraries directed against related rRNAs did not generate RNA ID signals, confirming the high selectivity of the approach (Figure.S14).

### RNA ID Assembly

RNA IDs were assembled using 5–10 μL of total RNA (5–100 ng/μL), 2.4 μL of oligopool (Integrated DNA Technologies; concentration of each oligonucleotide is 1 μM), 4 μL of 1 M LiCl, 4 μL of 10× TE (100 mM Tris–HCl pH 8.0, 10 mM EDTA), and 1 μL of 25 μM 3′-biotinylated imaging strand (IDT; 5̀-ACCACTAATGAGTGATATCC-TEG-biotin-3̀); nuclease-free water was added to a final volume of 40 μL.^26^ Samples were heated to 70 °C for 30 s, slowly cooled to 20 °C over 45 min, and stored at 4 °C or filtered immediately using a ProFlex PCR System (Applied Biosystems). The 40 μL mixture was mixed twice with 460 μL of washing buffer (10 mM Tris–HCl pH 8.0, 0.5 mM MgCl_2_) and centrifuged in 0.5 mL 100 kDa Amicon Ultra centrifugal filter (Sigma-Millipore, catalogue number UFC510008) at 9400 × *g* for 10 min. The filtered RNA IDs were retrieved by inverting the filter to a new 2 mL tube and centrifuged at 1000 × *g* for 2 min and stored at 4 °C prior to use. All buffers were filtered using Millipore 0.22 μm syringe filters. The samples are then diluted to 0.1 nM or to the respective concentration of RNA ID into a 4 M LiCl 1 × TE (pH 9.4) buffer solution immediately before the beginning of the nanopore measurement. The molarity for RNA targets in total RNA was estimated by assuming that 80% of total RNA mass is rRNA and that 23S, 16S, and 5S rRNAs are equimolar; their relative lengths were used to estimate the mass fraction of 16S or 23S rRNA and to calculate a correction factor for converting total RNA concentration (ng/μL) into 16S or 23S rRNA concentration.

### Short Heating Protocol for RNA ID Assembly

RNA ID was assembled using the identical procedure except the heating step as mentioned for RNA ID. The heating protocol was changed to 70, 85, 90, or 100 °C for 5 min and placed on ice for 5 min before ultrafiltration with Amicon 0.5 mL filter.

### DNA Carrier Assembly

The DNA constructs contained different “barcodes” plus a customizable overhang sequence that is a DNA version of the target RNA sequence. The DNA construct was synthesized from pairing a linearized 7.2 kbp single-stranded (ss) M13mp18 DNA (Guild Biosciences) and with 190 complementary oligonucleotides via Watson–Crick base pairing to produce full dsDNA for 1 h in a thermocycler. All oligonucleotides were synthesized by IDT and dissolved in IDTE (10 mM Tris–HCl, 0.1 mM EDTA, pH 8.0). The sequences are listed in Table S1.^52^ The sample is then filtered using a 100 kDa Amicon filter and measured with a Thermo Fisher Scientific NanoDrop 2000 spectrophotometer for concentration information. All samples were stored in a storage buffer of 10 mM Tris–HCl 0.5 mM MgCl_2_ after ultrafiltration. The design contains five groups of equally spaced simple dumbbell hairpin motifs within the 190 oligonucleotides to create the spikes, which act as a barcode on the DNA nanostructure. Each group consists of 11 DNA dumbbells to create a single spike.^52^ The sequences modified to contain DNA dumbbells are listed in Table S2. The overhang used for the target was created by replacing oligonucleotide nos. 142 and 143 from Table S1 with 50 bp target sequences as shown in Table S3.

### CRISPR-dCas9 and Streptavidin Binding Assay

Catalytically deactivated Cas9 D10A/H840A (dCas9) from*Streptococcus pyogenes*binds to a tracrRNA and a sequence-specific RNA (crRNA), both synthesized by Integrated DNA Technologies (IDT) and dissolved in IDTE (10 mM Tris–HCl, 0.1 mM EDTA, pH 8.0). The target sequences for the crRNA for the probes were designed using online software (http://chopchop.cbu.uib.no/). To assemble the dCas9 RNPs, first the tracrRNA (200 nM) was incubated at 90 °C for 2 min in a 1× low salt buffer (25 mM HEPES-NaOH (pH 8.0), 150 mM NaCl, 1 mM MgCl_2_) and then quickly cooled by placing the tube on ice. After 3 min, the dCas9 (100 nM) was added and incubated at 25 °C with tracrRNA for at least 5 min. After 5 min, crRNA (250 nM) was added and incubated for at least 10 min at 25 °C. Finally, the assembled dCas9 probes were then incubated with the DNA nanostructures or RNA IDs for at least 20 min at 25 °C, with the dCas9 probes added in excess of typically 15 dCas9 probes per DNA binding site. For the RNA IDs, after dCas9 binding, streptavidin was added in 10× excess per binding site directly before measurement and incubated at 25 °C for 10 min. The samples were then diluted to 0.1 nM or to the respective concentration of DNA nanostructure or RNA ID into a 2 M LiCl 1 × TE buffer (pH 8.0) solution immediately before the beginning of the nanopore measurement.

### Agarose Gel Electrophoresis

Our RNA ID constructs were run on a 0.8–1% (w/v) agarose gel (agarose for molecular biology, Sigma, catalogue number A9539), 1× TBE (0.089 M Tris–Borate, 0.002 M EDTA, pH 8.3; Sigma, catalogue number SRE0062) in an ice bath, for 1.5 h at 70 V. The gel was post-stained in 3× GelRed solution (Biotium, catalogue number 41001) for 10 min on a horizontal shaker. The stained gel was imaged with a GelDocIt­(UVP).

Gel images were analyzed using ImageJ (Fiji)^55^ by inverting grayscale and homogeneous background subtraction with a 100-pixel rolling ball.

### Nanopore Measurements

Conical nanopores with diameters of 10–15 nm were made of commercially available quartz capillaries (0.2 mm inner diameter/0.5 mm outer diameter Sutter Instruments, California). They were created using a laser-assisted pipet puller (P-2000, Sutter Instruments, California). Sixteen nanopores were then placed in a custom-templated polydimethylsiloxane (PDMS) chip containing a communal cis reservoir and individual trans-reservoirs. In order to generate the current, silver/silver chloride (Ag/AgCl) electrodes were connected to the cis- and trans-reservoirs in the PDMS chip. As only one nanopore can be measured at a time due to the electronics, the *trans* reservoir contains the electrode with 600 or 500 mV bias voltages (for dCas9 measurements), and the central *cis* reservoir is connected to ground.

An Axopatch 200B patch-clamp amplifier was used (Molecular Devices, California). The setup was operated in whole-cell mode with the internal filter set to 100 kHz. An 8-pole analogue low-pass Bessel filter (900CT, Frequency Devices, Illinois) with a cutoff frequency of 50 kHz was used to reduce noise. The applied voltage is controlled through an I/O analog-to-digital converter (DAQ cards, PCIe-6251, National Instruments, Texas). A LabVIEW program recorded the current signal at a bandwidth of 1 MHz.

### Nanopore Data Analysis

#### RNA IDs

Nanopore data analysis was performed from raw ionic current traces using a multistep filtering procedure.^20,49^ Candidate translocation events were first identified by requiring a minimum current drop from the open-pore baseline, excluding small background species and low-amplitude fluctuations. Minimum and maximum duration thresholds were then applied to remove short events from small molecules, proteins, or fragmented nucleic acids and long events arising from aggregates, pore interactions, or poorly defined complexes. Together, these filters enriched for intact RNA:DNA nanostructures.

As an additional validation step, we calculated the event charge deficit for each of the events. This time-integrated current blockade provides an orthogonal measure of molecule size and translocation behavior. Candidate RNA ID events were required to fall within the characteristic event charge deficit range expected for the corresponding RNA:DNA hybrid nanostructures, reducing false assignment beyond simple duration and amplitude thresholds.

RNA ID assignment was then performed on the filtered population of unfolded, linear events using the expected sequence of sublevels for classification. Because RNA:DNA hybrids translocate faster than double-stranded DNA of similar length,^51^ an assignment based solely on a fixed current threshold is not suitable. Instead, RNA IDs were identified by two hierarchical signal features: the current level corresponding to the linear RNA:DNA duplex and the pattern of additional downward current spikes produced by structural labels. The number, order, and relative spacing of these label-associated sublevels define the RNA ID signature. Thus, RNA IDs were assigned from the combined presence of the RNA:DNA duplex level and the expected sequence of structural label subevents.

All measurements were repeated in independent replicate experiments. For each condition, we report the event frequency of RNA IDs and translocation times. Measurement-specific parameters, including buffer composition, pH, and open-pore current, were tabulated for all data sets (Table S16). The typical frequency of events varied between nanopores, ranging from 5 to 20 per minute. The event frequency depends on the nanopore geometry and shape.^66^

#### dCas9

For data using DNA carrier and dCas9, the following procedure was followed. First, a translocation finder Python script (https://gitlab.com/keyserlab/nanopyre) was used to identify the events from the raw traces using user-defined thresholds (minimum 0.1 ms duration, minimum 0.1 nA current drop) and stores them in an hdf5 file. Next, the hdf5 file is loaded into the GUI categorizer Python script, found here: https://gitlab.com/keyserlab/nanopycker. Events were then sorted based on the number of peaks or the barcodes and the presence or absence of proteins bound.

The binding of dCas9 to RNA:DNA *Salmonella* samples:

$$\%{\mathrm{events}}_{3\mathrm{peaks}}=\frac{N_{3\mathrm{peaks}}}{N_{3\mathrm{peaks}}+N_{2\mathrm{peaks}}}\times 100$$

where three peaks correspond to “22C” ID and two peaks correspond to “22” ID. Events containing one peak or no peaks were discarded.

The specificity as seen in Figure S11 was calculated using the following formula:

$$\%\mathrm{dCas}9{\mathrm{Events}}_{11111}=\frac{N_{11111\mathrm{dCas}9}}{N_{11111\mathrm{dCas}9}+N_{11001\mathrm{dCas}9}}\times 100$$

$$\%\mathrm{dCas}9{\mathrm{Events}}_{11001}=\frac{N_{11001\mathrm{dCas}9}}{N_{11111\mathrm{dCas}9}+N_{11001\mathrm{dCas}9}}\times 100$$

## Supplementary Material



## Notes

RNA ID nanopore events; multiplexed detection of bacterial targets and MS2 and M13 bacteriophage nucleic acids; validation of rRNA ID assembly specificity by nanopore readout; *Salmonella enterica* serovar 16S rRNA sequence alignment; uninterrupted raw ionic current traces; experimental nanopore ionic current data analysis workflow; oligonucleotide sequences used for RNA ID assembly; and nanopore measurement details, including buffer composition, pH, open-pore current, RNA ID event frequency, and mean translocation time (PDF)
