# Supplementary material for: Modular RNA:DNA Nanostructures Enable Nanopore Profiling of rRNA Processing and rRNA Variants
Source: ACS Nano. 2026 May 13;20(20):14634–44. doi: 10.1021/acsnano.6c01132 (PMC13218044; doi:10.1021/acsnano.6c01132)
Supplement: Supplementary file 1 [file nn6c01132_si_001.pdf]

Supporting Information for

Modular RNA:DNA nanostructures enable nanopore profiling of ribosomal RNA processing and rRNA variants

Filip Boskovic[1,#], Sarah Sandler[1,#], Simon Brauburger[1], Yuan Shui[1], Bhavik Kumar[1], Joana Pereira Dias[2,3], Plamena Naydenova[2,3], Jinbo Zhu[1], Stephen Baker[4], Ulrich F. Keyser[1,*]

[1]Cavendish Laboratory, University of Cambridge, Cambridge CB3 0HE, UK

[2]University of Cambridge School of Clinical Medicine, Cambridge Biomedical Campus, Hills Road, Cambridge CB2 0SP, UK

[3]Department of Medicine, University of Cambridge School of Clinical Medicine, Cambridge Biomedical Campus, Hills Road, Cambridge CB2 0SP, UK

[4]A*STAR Infectious Diseases Labs (A*STAR IDL), Agency for Science, Technology and Research (A*STAR), Singapore 138648, Singapore

[#]Co-first authors

*Correspondence to: ufk20@cam.ac.uk

This document includes

Figs. S1 to S14
Tables S1 to S16

Fig.S1

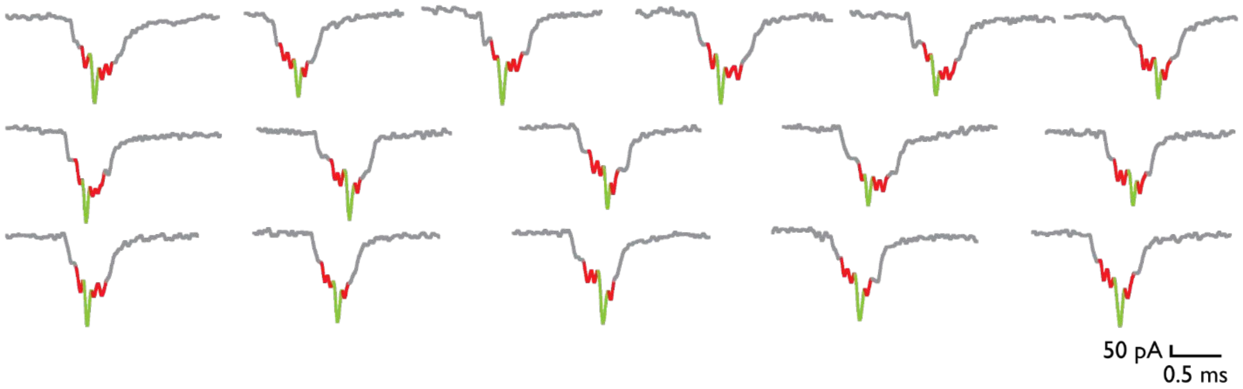

Fig.S1 Typical nanopore events for *E.coli* J01859.1 16S rRNA ID `1131`.

Fig.S2

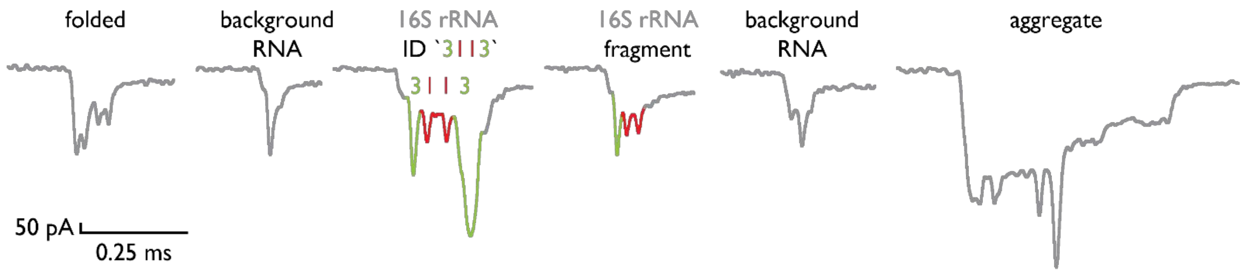

Fig.S2 Events from nanopore measurements obtained during a 10-min measurement from *Salmonella* Typhimurium. Specificity is shown by selected background events with highlighted additional 16S rRNA ID `3113` events or fragments.

Fig.S3

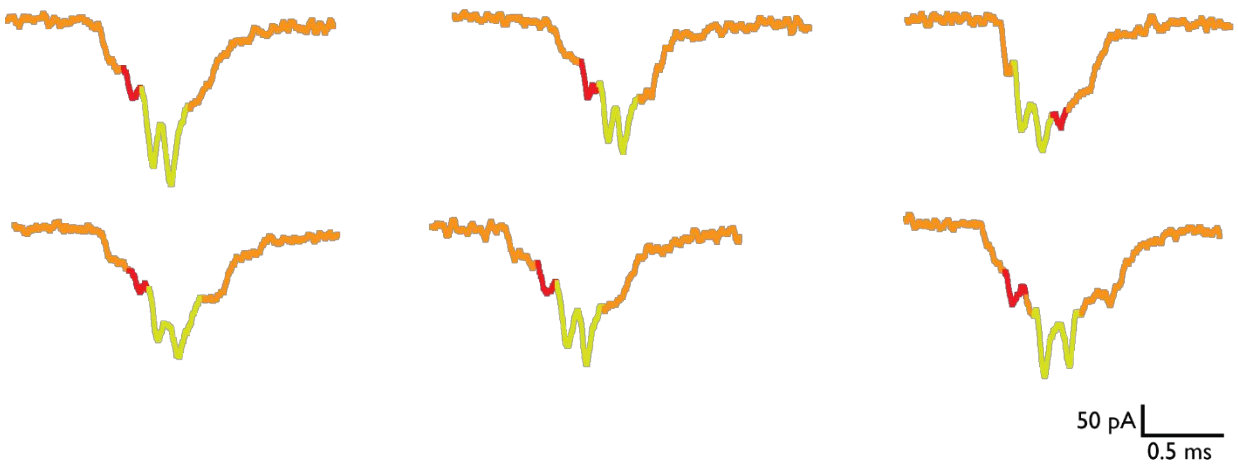

Fig.S3 Nanopore events for *A. baumannii* 16S rRNA ID `0331`.

Fig.S4

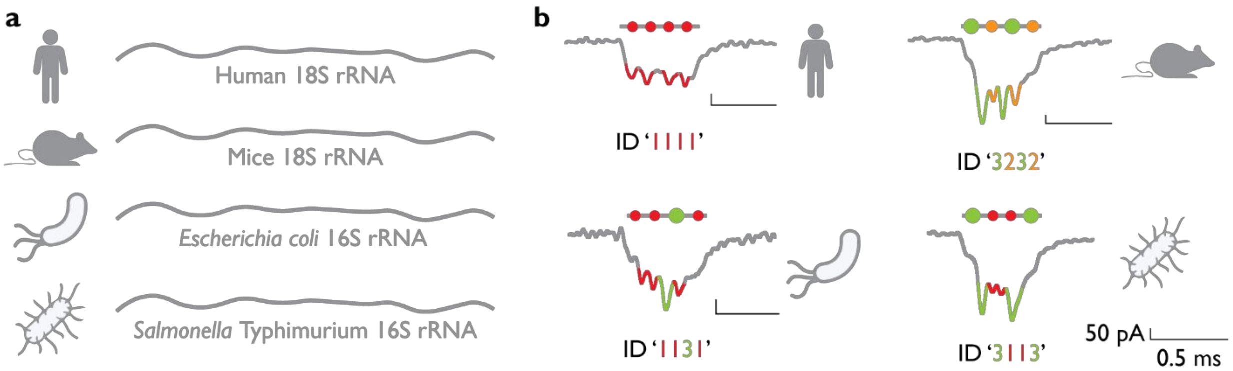

Fig.S4 Identifiers enable multiplexed species determination using 16S or 18S rRNA and bacterial or viral RNA. (**a**) Total RNA samples from four different sources, humans, mice, E. coli, and S. Typhimurium, were isolated and targeted for either 18S or 16S rRNAs. (**b**) RNA ID designs and their corresponding nanopore readouts are presented to showcase the capability for parallel identification of multiple organisms.

Fig.S5

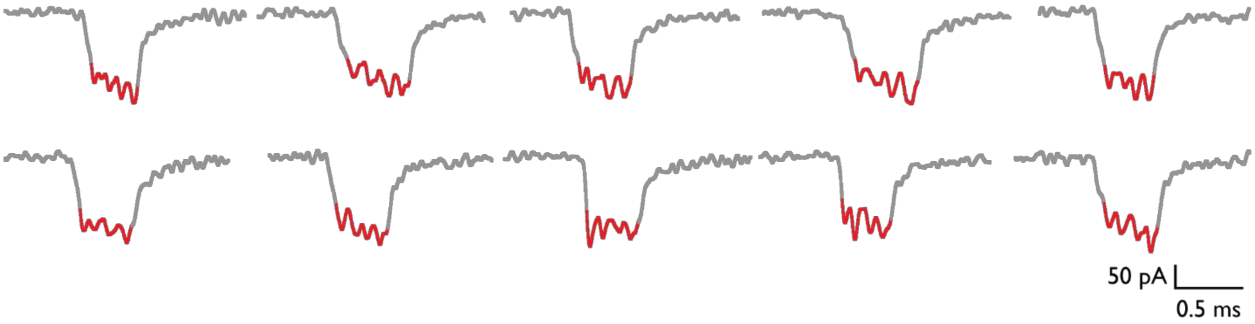

Fig.S5 Nanopore events for human 18S rRNA ID `1111`.

Fig.S6

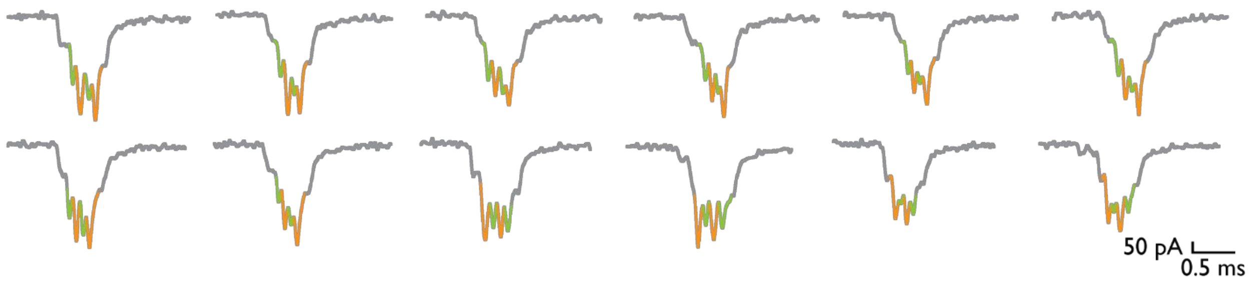

Fig.S6 Nanopore events for mice 18S rRNA ID `3232`.

Fig.S7

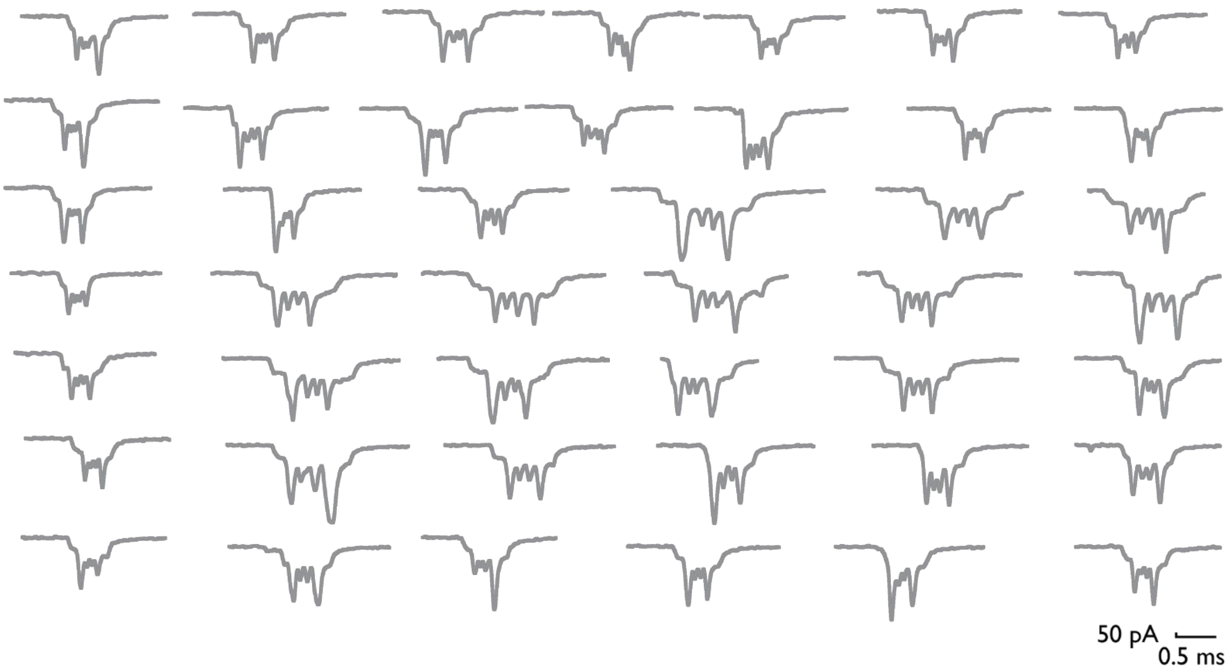

Fig.S7 Nanopore events for *S.*Typhimurium 16S rRNA ID `3113`.

Fig.S8

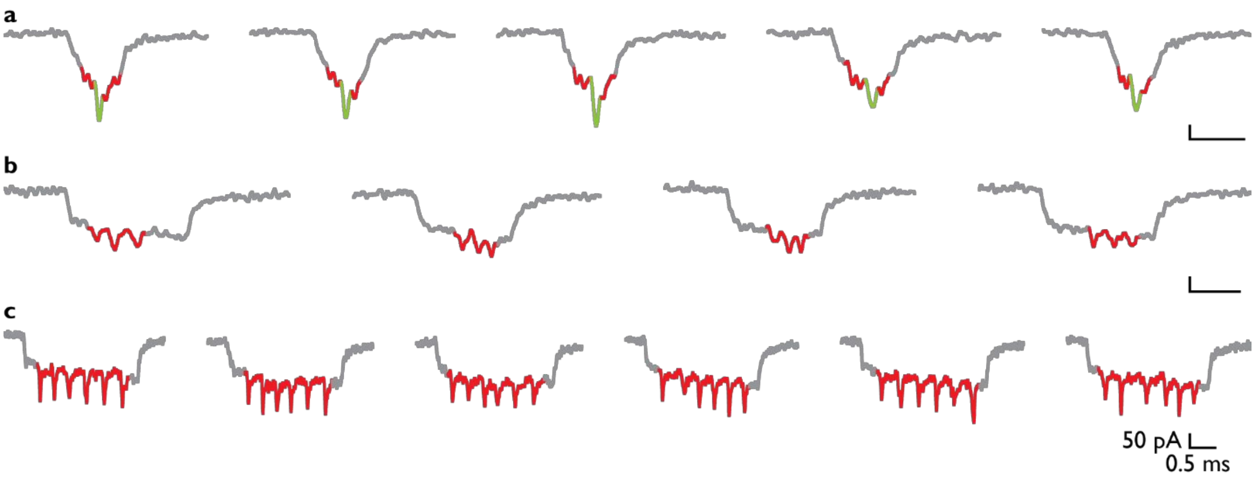

Fig.S8 Nanopore events for *E.coli* coinfection with single-stranded M13 DNA phage (7,228 nt) and single-stranded MS2 RNA phage (3,569 nt). Besides *E.coli* 16S rRNA ID `1131`, we created M13 ID `111111` and MS2 ID `111`.

Fig.S9

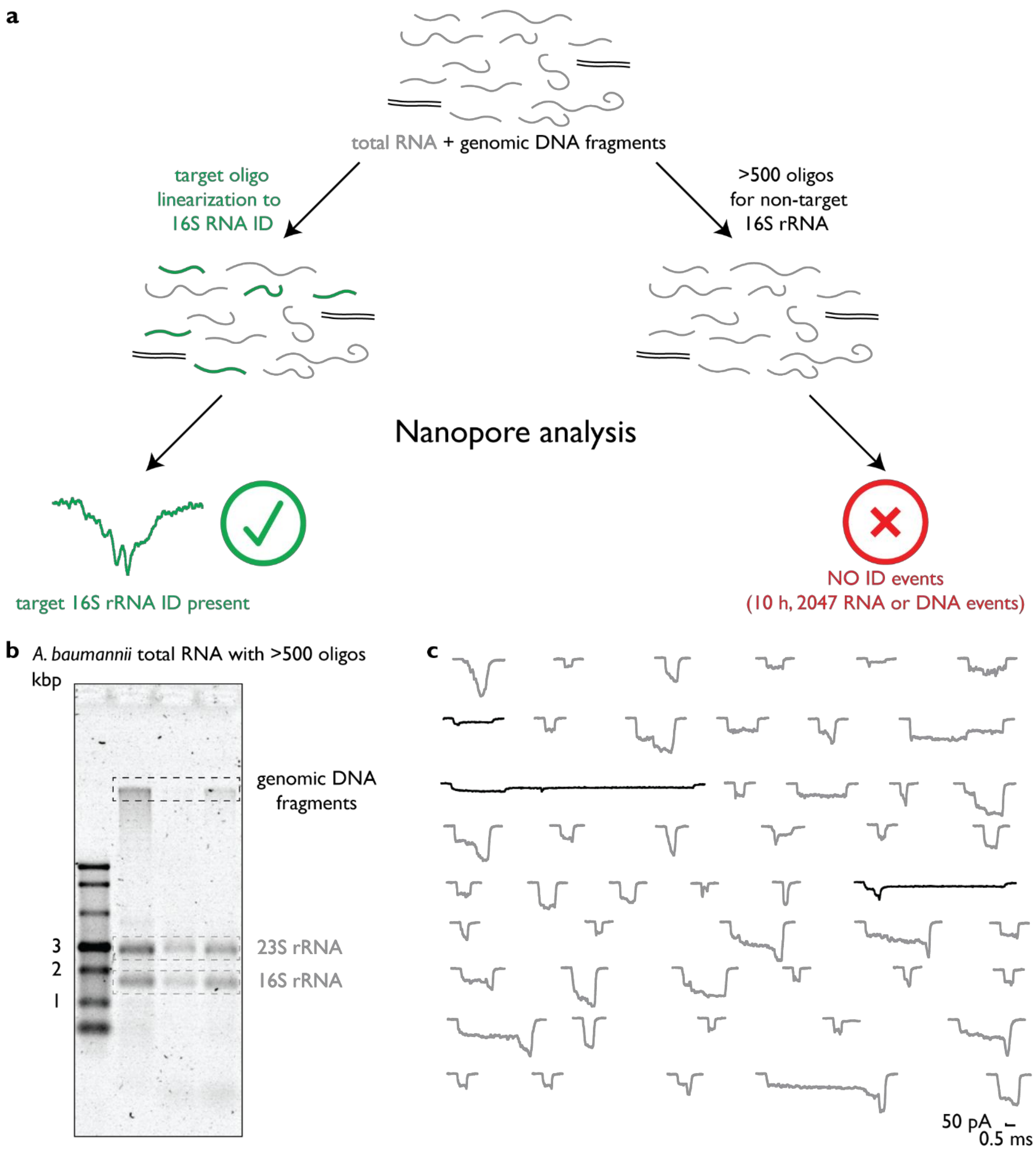

Fig.S9 Testing for non-specific 16S rRNA ID assembly using total RNA from *A. baumannii*. (**a**) Procedure for assembly RNA IDs and their nanopore analysis is schematically presented. Using the correct oligonucleotides targeting 16S rRNA *A. baumannii,* we identified RNA ID `133` (as shown in Fig. 4). We tested non-specific RNA ID assembly by mixing almost five hundred oligonucleotides targeting human 18S rRNA, mice 18S rRNA, *E. coli* 16S rRNA, *Salmonella* spp.

16S and 23S RNA, but by using total RNA from *A. baumannii*. After a 10-h nanopore measurement, we detected 2047 nanopore events originating from background RNA and DNA while none of these had the designed RNA ID. (**b**) 0.8% (v/v) agarose gel electrophoresis shows that there is no shift of 16S rRNA after assembly with >500 oligonucleotides. Lane 1 – ssRNA ladder (NEB, catalogue no. N0362S); Lane 2 – total RNA of KL49 *A. baumannii*; Lane 3 – total RNA of KL49 *A. baumannii* assembled with 500 oligonucleotides; Lane 4 – total RNA of control *A. baumannii* assembled with 500 oligonucleotides. (**c**) 50 consecutive nanopore events when >500 oligonucleotides were used with total RNA. RNA blob-like events are shown in grey, and genomic DNA fragments are shown in black. None of these events fulfill requirements for positive RNA ID identification for nanopore events.

Fig.S10

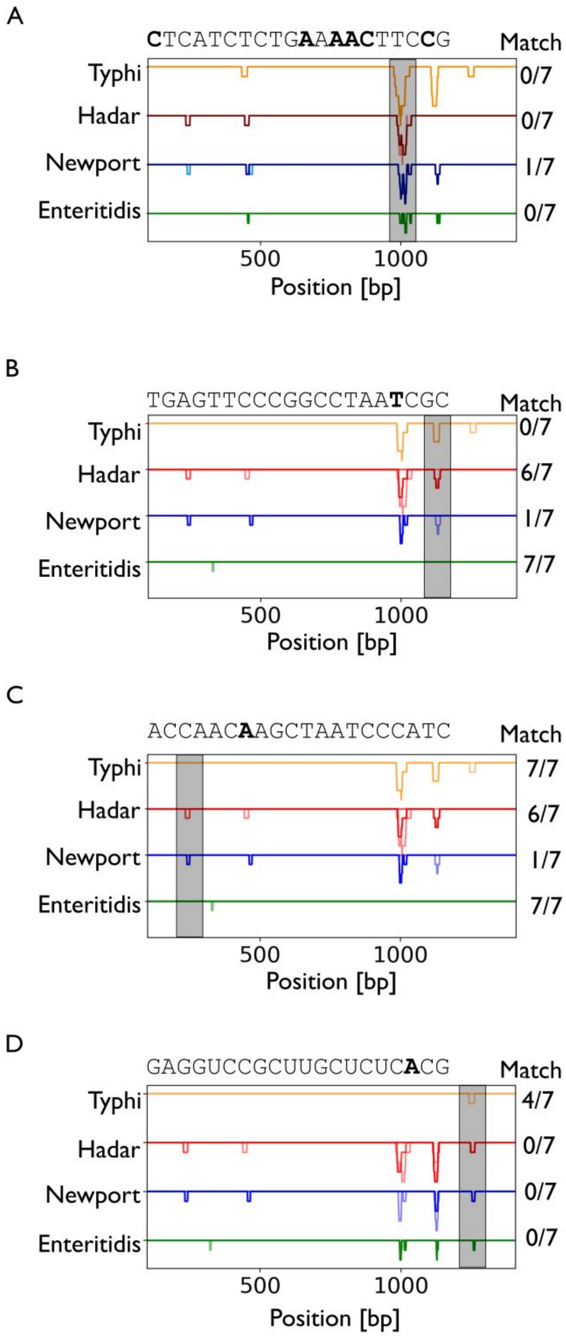

Fig.S10 Sequence alignments for several locations along rRNA sequences from *Salmonella enterica* serovars. Per bacterium there are seven potential rRNA transcripts originating from seven rRNA genes. Match fraction represents how many sequences the designed guide RNA matches for within the 7 different rRNA sequences.

Fig.S11

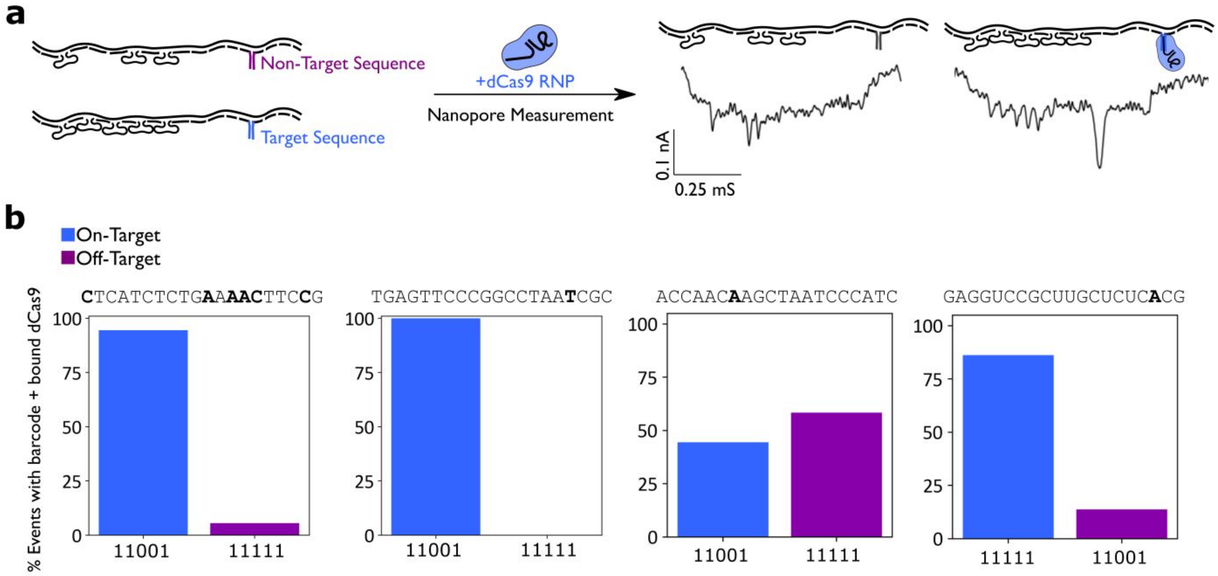

Fig.S11 Specificity of several probes for target regions to identify single-base pair mismatches. (A) Examples of dCas9 (in blue) bound and unbound nanopore events. (B-D) Specificity of different probes to target sequence developed using method by Sandler et al.[1].

Fig.S12

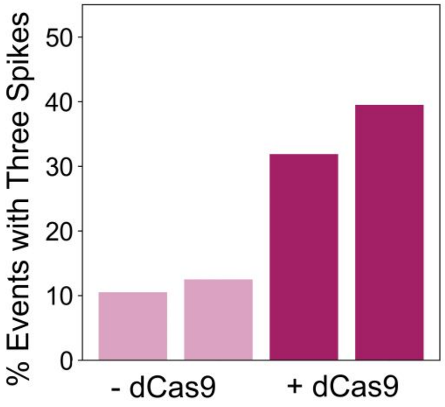

Fig.S12 Repeats for *S.* Typhi sample. Samples were assembled independently and measured both in the absence (Sample 1, N=25 linear nanopore events, Sample 2, N=16 linear nanopore events) and presence of dCas9 (Sample 1, N=69 linear nanopore events, Sample 2, N=33 linear nanopore events).

Fig.S13

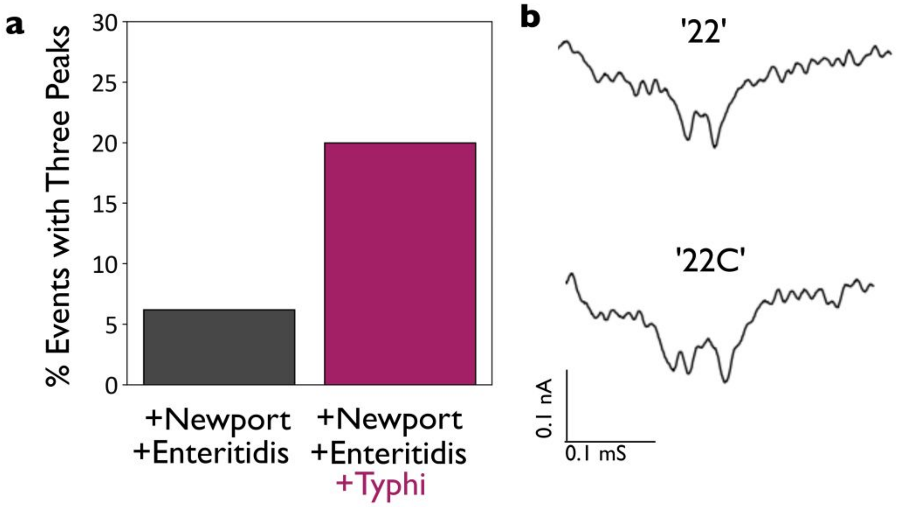

Fig.S13 *S*. Typhi can be differentiated in samples with mixed strains of *S*. Newport and *S*. Enteritidis using dCas9 by an increase in nanopore events observed with three peaks when *S*. Typhi RNA is mixed in with *S*. Newport and *S*. Enteritidis (**a**). An event with no dCas9 bound (two peaks) is likely *S*. Newport or *S*. Enteritidis (top) and event with dCas9 bound is likely *S*. Typhi (**b**).

Fig.S14

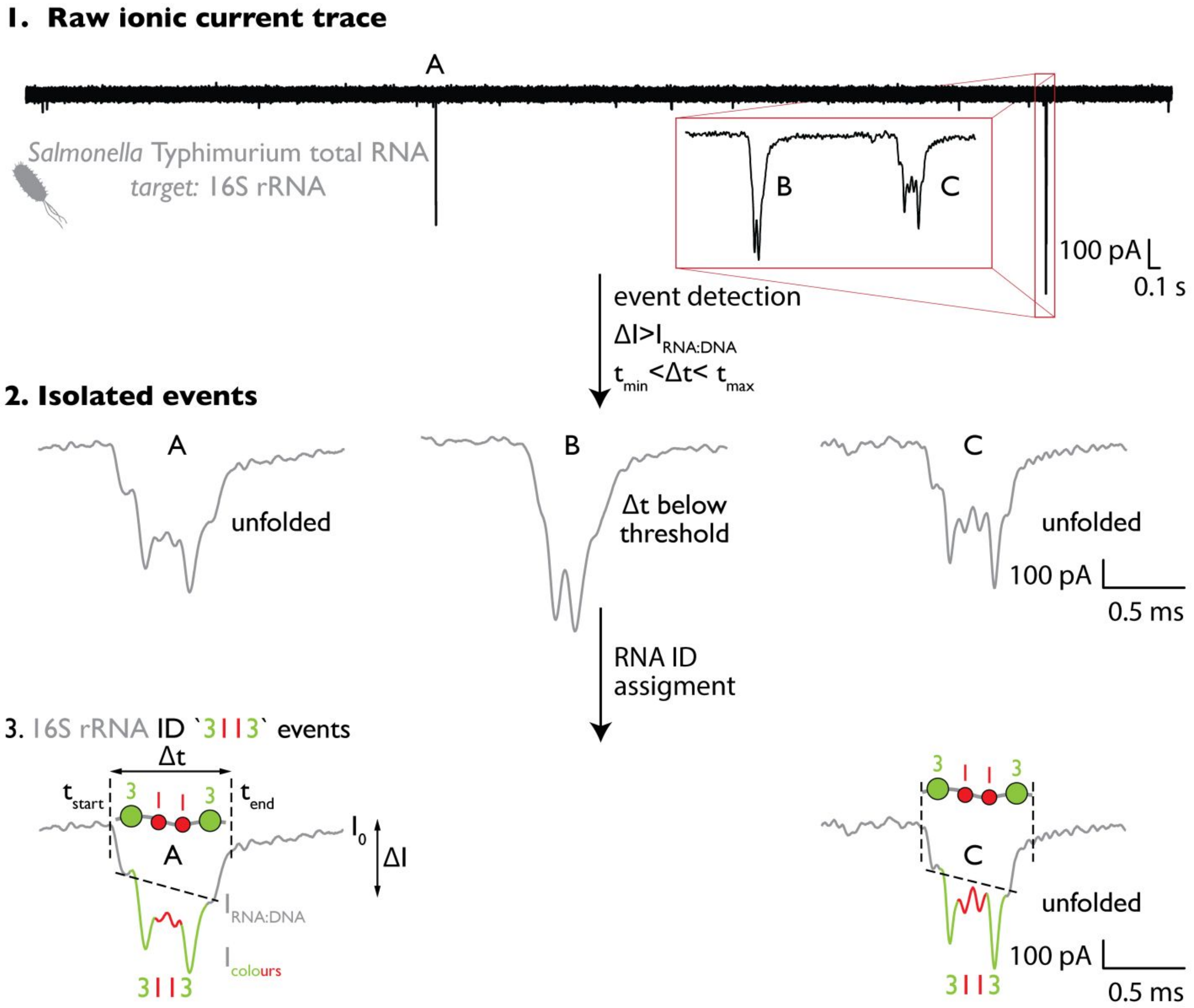

Fig.S14. Uninterrupted raw ionic current trace of the 16S *S. Typhimurium* RNA ID '3113'. Nanopore events were first identified (for this trace are labelled as A, B, and C) using thresholds for current blockade, event duration, and event charge deficit, adjusted for each measurement, to exclude fragments, aggregates, and short non-specific events. The resulting events were then filtered by residence time (Δt) to remove folded translocation events. RNA IDs were subsequently assigned on the basis of the expected target ID design, and each event was assessed for the presence of the correct number of sites and the relative size of the structural labels. I_0 denotes the open-pore current.

Table S1

Table S1. The core DNA oligonucleotides are complementary to linearized ssM13mp18 (7228 nt).

Oligonucleotide Number	Sequence (5′→3′)	Oligonucleotide Number	Sequence (5′→3′)
1	TTTTCGTAATCATGGTCATAGCTGTTTCCTGTGTGAAATTGTTATC	96	CTTGAGCCATTTGGGAATTAGAGCCAGCAAAATCACCA
2	CGCTCACAATTCCACACAACATACGAGCCGGAAGCATA	97	GTAGCACCATTACCATTAGCAAGGCCGGAAACGTCACC
3	AAGTGTAAAGCCTGGGGTGCCTAATGAGTGAGCTAACT	98	AATGAAACCATCGATAGCAGCACCGTAATCAGTAGCGA
4	CACATTAATTGCGTTGCGCTCACTGCCCGCTTTCCAGT	99	CAGAATCAAGTTTGCCTTTAGCGTCAGACTGTAGCGCG
5	CGGGAAACCTGTCGTGCCAGCTGCATTAATGAATCGGC	100	TTTTCATCGGCATTTTCGGTCATAGCCCCCTTATTAGC
6	CAACGCGCGGGGAGAGGCGGTTTGCGTATTGGGCGCCA	101	GTTTGCCATCTTTTCATAATCAAAATCACCGGAACCAG
7	GGGTGGTTTTTCTTTTCACCAGTGAGACGGGCAACAGC	102	AGCCACCACCGGAACCGCCTCCCTCAGAGCCGCCACCC
8	TGATTGCCCTTCACCGCCTGGCCCTGAGAGAGTTGCAG	103	TCAGAACCGCCACCCTCAGAGCCACCACCCTCAGAGCC
9	CAAGCGGTCCACGCTGGTTTGCCCCAGCAGGCGAAAAT	104	GCCACCAGAACCACCACCAGAGCCGCCGCCAGCATTGA
10	CCTGTTTGATGGTGGTTCCGAAATCGGCAAAATCCCTT	105	CAGGAGGTTGAGGCAGGTCAGACGATTGGCCTTGATAT
11	ATAAATCAAAAGAATAGCCCGAGATAGGGTTGAGTGTT	106	TCACAAACAAATAAATCCTCATTAAAGCCAGAATGGAA
12	GTTCCAGTTTGGAACAAGAGTCCACTATTAAAGAACGT	107	AGCGCAGTCTCTGAATTTACCGTTCCAGTAAGCGTCAT
13	GGACTCCAACGTCAAAGGGCGAAAAACCGTCTATCAGG	108	ACATGGCTTTTGATGATACAGGAGTGTACTGGTAATAA
14	GCGATGGCCCACTACGTGAACCATCACCCAAATCAAGT	109	GTTTTAACGGGGTCAGTGCCTTGAGTAACAGTGCCCGT
15	TTTTTGGGGTCGAGGTGCCGTAAAGCACTAAATCGGAA	110	ATAAACAGTTAATGCCCCCTGCCTATTTCGGAACCTAT
16	CCCTAAAGGGAGCCCCCGATTTAGAGCTTGACGGGGAA	111	TATTCTGAAACATGAAAGTATTAAGAGGCTGAGACTCC
17	AGCCGGCGAACGTGGCGAGAAAGGAAGGGAAGAAAGCG	112	TCAAGAGAAGGATTAGGATTAGCGGGGTTTTGCTCAGT
18	AAAGGAGCGGGCGCTAGGGCGCTGGCAAGTGTAGCGGT	113	ACCAGGCGGATAAGTGCCGTCGAGAGGGTTGATATAAG
19	CACGCTGCGCGTAACCACCACACCCGCCGCGCTTAATG	114	TATAGCCCGGAATAGGTGTATCACCGTACTCAGGAGGT
20	CGCCGCTACAGGGCGCGTACTATGGTTGCTTTGACGAG	115	TTAGTACCGCCACCCTCAGAACCGCCACCCTCAGAACC
21	CACGTATAACGTGCTTTCCTCGTTAGAATCAGAGCGGG	116	GCCACCCTCAGAGCCACCACCCTCATTTTCAGGGATAG
22	AGCTAAACAGGAGGCCGATTAAAGGGATTTTAGACAGG	117	CAAGCCCAATAGGAACCCATGTACCGTAACACTGAGTT
23	AACGGTACGCCAGAATCCTGAGAAGTGTTTTTATAATC	118	TCGTCACCAGTACAAACTACAACGCCTGTAGCATTCCA
24	AGTGAGGCCACCGAGTAAAAGAGTCTGTCCATCACGCA	119	CAGACAGCCCTCATAGTTAGCGTAACGATCTAAAGTTT
25	AATTAACCGTTGTAGCAATACTTCTTTGATTAGTAATA	120	TGTCGTCTTTCCAGACGTTAGTAAATGAATTTTCTGTA
26	ACATCACTTGCCTGAGTAGAAGAACTCAAACTATCGGC	121	TGGGATTTTGCTAAACAACTTTCAACAGTTTCAGCGGA
27	CTTGCTGGTAATATCCAGAACAATATTACCGCCAGCCA	122	GTGAGAATAGAAAGGAACAACTAAAGGAATTGCGAATA
28	TTGCAACAGGAAAAACGCTCATGGAAATACCTACATTT	123	ATAATTTTTTCACGTTGAAAATCTCCAAAAAAAAGGCT
29	TGACGCTCAATCGTCTGAAATGGATTATTTACATTGGC	124	CCAAAAGGAGCCTTTAATTGTATCGGTTTATCAGCTTG
30	AGATTCACCAGTCACACGACCAGTAATAAAAGGACAT	125	CTTTCGAGGTGAATTTCTTAAACAGCTTGATACCGATA

31	TCTGGCCAACAGAGATAGAACCCTTCTGACCTGAAAGC	126	GTTGCGCCGACAATGACAACAACCATCGCCCACGCATA
32	GTAAGAATACGTGGCACAGACAATATTTTTGAATGGCT	127	ACCGATATATTCGGTCGCTGAGGCTTGCAGGGAGTTAA
33	ATTAGTCTTTAATGCGCGAACTGATAGCCCTAAAACAT	128	AGGCCGCTTTTGCGGGATCGTCACCCTCAGCAGCGAAA
34	CGCCATTAAAAATACCGAACGAACCACCAGCAGAAGAT	129	GACAGCATCGGAACGAGGGTAGCAACGGCTACAGAGGC
35	AAAACAGAGGTGAGGCGGTCAGTATTAACACCGCCTGC	130	TTTGAGGACTAAAGACTTTTTCATGAGGAAGTTTCCAT
36	AACAGTGCCACGCTGAGAGCCAGCAGCAAATGAAAAAT	131	TAAACGGGTAAAATACGTAATGCCACTACGAAGGCACC
37	CTAAAGCATCACCTTGCTGAACCTCAAATATCAAACCC	132	AACCTAAAACGAAAGAGGCAAAAGAATACACTAAAACA
38	TCAATCAATATCTGGTCAGTTGGCAAATCAACAGTTGA	133	CTCATCTTTGACCCCCAGCGATTATACCAAGCGCGAAA
39	AAGGAATTGAGGAAGGTTATCTAAAATATCTTTAGGAG	134	CAAAGTACAACGGAGATTTGTATCATCGCCTGATAAAT
40	CACTAACAACTAATAGATTAGAGCCGTCAATAGATAAT	135	TGTGTCGAAATCCGCGACCTGCTCCATGTTACTTAGCC
41	ACATTTGAGGATTTAGAAGTATTAGACTTTACAAACAA	136	GGAACGAGGCGCAGACGGTCAATCATAAGGGAACCGAA
42	TTCGACAACTCGTATTAAATCCTTTGCCCGAACGTTAT	137	CTGACCAACTTTGAAAGAGGACAGATGAACGGTGTACA
43	TAATTTTAAAAGTTTGAGTAACATTATCATTTTGCGGA	138	GACCAGGCGCATAGGCTGGCTGACCTTCATCAAGAGTA
44	ACAAAGAAACCACCAGAAGGAGCGGAATTATCATCATA	139	ATCTTGACAAGAACCGGATATTCATTACCCAAATCAAC
45	TTCCTGATTATCAGATGATGGCAATTCATCAATATAAT	140	GTAACAAAGCTGCTCATTCAGTGAATAAGGCTTGCCCT
46	CCTGATTGTTTGGATTATACTTCTGAATAATGGAAGGG	141	GACGAGAAACACCAGAACGAGTAGTAAATTGGGCTTGA
47	TTAGAACCTACCATATCAAAATTATTTGCACGTAAAAC	142	GATGGTTTAATTTCAACTTTAATCATTGTGAATTACCT
48	AGAAATAAAGAAATTGCGTAGATTTTCAGGTTTAACGT	143	TATGCGATTTTAAGAACTGGCTCATTATACCAGTCAGG
49	CAGATGAATATACAGTAACAGTACCTTTTACATCGGGA	144	ACGTTGGGAAGAAAAATCTACGTTAATAAAACGAACTA
50	GAAACAATAACGGATTCGCCTGATTGCTTTGAATACCA	145	ACGGAACAACATTATTACAGGTAGAAAGATTCATCAGT
51	AGTTACAAAATCGCGCAGAGGCGAATTATTCATTTCAA	146	TGAGATTTAGGAATACCACATTCAACTAATGCAGATAC
52	TTACCTGAGCAAAAGAAGATGATGAAACAAACATCAAG	147	ATAACGCCAAAAGGAATTACGAGGCATAGTAAGAGCAA
53	AAAACAAAATTAATTACATTTAACAATTTCATTTGAAT	148	CACTATCATAACCCTCGTTTACCAGACGACGATAAAAA
54	TACCTTTTTTAATGGAAACAGTACATAAATCAATATAT	149	CCAAAATAGCGAGAGGCTTTTGCAAAAGAAGTTTTGCC
55	GTGAGTGAATAACCTTGCTTCTGTAAATCGTCGCTATT	150	AGAGGGGGTAATAGTAAAATGTTTAGACTGGATAGCGT
56	AATTAATTTTCCCTTAGAATCCTTGAAAACATAGCGAT	151	CCAATACTGCGGAATCGTCATAAATATTCATTGAATCC
57	AGCTTAGATTAAGACGCTGAGAAGAGTCAATAGTGAAT	152	CCCTCAAATGCTTTAAACAGTTCAGAAAACGAGAATGA
58	TTATCAAAATCATAGGTCTGAGAGACTACCTTTTTAAC	153	CCATAAATCAAAAATCAGGTCTTTACCCTGACTATTAT
59	CTCCGGCTTAGGTTGGGTTATATAACTATATGTAAATG	154	AGTCAGAAGCAAAGCGGATTGCATCAAAAAGATTAAGA
60	CTGATGCAAATCCAATCGCAAGACAAAGAACGCGAGAA	155	GGAAGCCCGAAAGACTTCAAATATCGCGTTTTAATTCG
61	AACTTTTTCAAATATATTTTAGTTAATTTCATCTTCTG	156	AGCTTCAAAGCGAACCAGACCGGAAGCAAACTCCAACA
62	ACCTAAATTTAATGGTTTGAAATACCGACCGTGTGATA	157	GGTCAGGATTAGAGAGTACCTTTAATTGCTCCTTTTGA
63	AATAAGGCGTTAAATAAGAATAAACACCGGAATCATAA	158	TAAGAGGTCATTTTTGCGGATGGCTTAGAGCTTAATTG
64	TTACTAGAAAAAGCCTGTTTAGTATCATATGCGTTATA	159	CTGAATATAATGCTGTAGCTCAACATGTTTTAAATATG
65	CAAATTCTTACCAGTATAAAGCCAACGCTCAACAGTAG	160	CAACTAAAGTACGGTGTCTGGAAGTTTCATTCCATATA
66	GGCTTAATTGAGAATCGCCATATTTAACAACGCCAACA	161	ACAGTTGATTCCCAATTCTGCGAACGAGTAGATTTAGT

67	TGTAATTTAGGCAGAGGCATTTTCGAGCCAGTAATAAG	162	TTGACCATTAGATACATTTCGCAAATGGTCAATAACCT
68	AGAATATAAAGTACCGACAAAAGGTAAAGTAATTCTGT	163	GTTTAGCTATATTTTCATTTGGGGCGCGAGCTGAAAAG
69	CCAGACGACGACAATAAACAACATGTTCAGCTAATGCA	164	GTGGCATCAATTCTACTAATAGTAGTAGCATTAACATC
70	GAACGCGCCTGTTTATCAACAATAGATAAGTCCTGAAC	165	CAATAAATCATACAGGCAAGGCAAAGAATTAGCAAAAT
71	AAGAAAAATAATATCCCATCCTAATTTACGAGCATGTA	166	TAAGCAATAAAGCCTCAGAGCATAAAGCTAAATCGGTT
72	GAAACCAATCAATAATCGGCTGTCTTTCCTTATCATTC	167	GTACCAAAAACATTATGACCCTGTAATACTTTTGCGGG
73	CAAGAACGGGTATTAAACCAAGTACCGCACTCATCGAG	168	AGAAGCCTTTATTTCAACGCAAGGATAAAAATTTTTAG
74	AACAAGCAAGCCGTTTTTATTTTCATCGTAGGAATCAT	169	AACCCTCATATATTTTAAATGCAATGCCTGAGTAATGT
75	TACCGCGCCCAATAGCAAGCAAATCAGATATAGAAGGC	170	GTAGGTAAAGATTCAAAAGGGTGAGAAAGGCCGGAGAC
76	TTATCCGGTATTCTAAGAACGCGAGGCGTTTTAGCGAA	171	AGTCAAATCACCATCAATATGATATTCAACCGTTCTAG
77	CCTCCCGACTTGCGGGAGGTTTTGAAGCCTTAAATCAA	172	CTGATAAATTAATGCCGGAGAGGGTAGCTATTTTTGAG
78	GATTAGTTGCTATTTTGCACCCAGCTACAATTTTATCC	173	AGATCTACAAAGGCTATCAGGTCATTGCCTGAGAGTCT
79	TGAATCTTACCAACGCTAACGAGCGTCTTTCCAGAGCC	174	GGAGCAAACAAGAGAATCGATGAACGGTAATCGTAAAA
80	TAATTTGCCAGTTACAAAATAAACAGCCATATTATTTA	175	CTAGCATGTCAATCATATGTACCCCGGTTGATAATCAG
81	TCCCAATCCAAATAAGAAACGATTTTTTGTTTAACGTC	176	AAAAGCCCCAAAAACAGGAAGATTGTATAAGCAAATAT
82	AAAAATGAAAATAGCAGCCTTTACAGAGAGAATAACAT	177	TTAAATTGTAAACGTTAATATTTTGTTAAAATTCGCAT
83	AAAAACAGGGAAGCGCATTAGACGGGAGAATTAACTGA	178	TAAATTTTTGTTAAATCAGCTCATTTTTTAACCAATAG
84	ACACCCTGAACAAAGTCAGAGGGTAATTGAGCGCTAAT	179	GAACGCCATCAAAAATAATTCGCGTCTGGCCTTCCTGT
85	ATCAGAGAGATAACCCACAAGAATTGAGTTAAGCCCAA	180	AGCCAGCTTTCATCAACATTAAATGTGAGCGAGTAACA
86	TAATAAGAGCAAGAAACAATGAAATAGCAATAGCTATC	181	ACCCGTCGGATTCTCCGTGGGAACAAACGGCGGATTGA
87	TTACCGAAGCCCTTTTTAAGAAAAGTAAGCAGATAGCC	182	CCGTAATGGGATAGGTCACGTTGGTGTAGATGGGCGCA
88	GAACAAAGTTACCAGAAGGAAACCGAGGAAACGCAATA	183	TCGTAACCGTGCATCTGCCAGTTTGAGGGGACGACGAC
89	ATAACGGAATACCCAAAAGAACTGGCATGATTAAGACT	184	AGTATCGGCCTCAGGAAGATCGCACTCCAGCCAGCTTT
90	CCTTATTACGCAGTATGTTAGCAAACGTAGAAAATACA	185	CCGGCACCGCTTCTGGTGCCGGAAACCAGGCAAAGCGC
91	TACATAAAGGTGGCAACATATAAAAGAAACGCAAAGAC	186	CATTCGCCATTCAGGCTGCGCAACTGTTGGGAAGGGCG
92	ACCACGGAATAAGTTTATTTTGTCACAATCAATAGAAA	187	ATCGGTGCGGGCCTCTTCGCTATTACGCCAGCTGGCGA
93	ATTCATATGGTTTACCAGCGCCAAAGACAAAAGGGCGA	188	AAGGGGGATGTGCTGCAAGGCGATTAAGTTGGGTAACG
94	CATTCAACCGATTGAGGGAGGGAAGGTAAATATTGACG	189	CCAGGGTTTTCCCAGTCACGACGTTGTAAAACGACGGC
95	GAAATTATTCATTAAAGGTGAATTATCACCGTCACCGA	190	CAGTGCCAAGCTTGCATGCCTGCAGGTCGACTCTAGAGGATCTTTT

Table S2

Table S2. Barcode ‘bits’ for DNA nanostructure with ´11111´ and one overhang as shown in Fig. 4a. The following replacements are made to create the ´1´ bits in the barcode portion of the nanostructure.

Replaced oligonucleotides from Table S1.	Sequence (5′→3′)
**For the first bit:**<br><br>26, 27, 28, 29, 30, 31 and 32	CTGAAAGCGTAAGAATACGTGGCACAGACAATATTTTTGAATGGCT
	ACATCACTTGTCCTCTTTTGAGGAACAAGTTTTCTTGTCCTGAGTAGA
	AGAACTCAAATCCTCTTTTGAGGAACAAGTTTTCTTGTCTATCGGCCT
	TGCTGGTAATTCCTCTTTTGAGGAACAAGTTTTCTTGTATCCAGAACA
	ATATTACCGCTCCTCTTTTGAGGAACAAGTTTTCTTGTCAGCCATTGC
	AACAGGAAAATCCTCTTTTGAGGAACAAGTTTTCTTGTACGCTCATGG
	AAATACCTACTCCTCTTTTGAGGAACAAGTTTTCTTGTATTTTGACGC
	TCAATCGTCTTCCTCTTTTGAGGAACAAGTTTTCTTGTGAAATGGATT
	ATTTACATTGTCCTCTTTTGAGGAACAAGTTTTCTTGTGCAGATTCAC
	CAGTCACACGTCCTCTTTTGAGGAACAAGTTTTCTTGTACCAGTAATA
	AAAGGGACATTCCTCTTTTGAGGAACAAGTTTTCTTGTTCTGGCCAAC
	AGAGATAGAATCCTCTTTTGAGGAACAAGTTTTCTTGTCCCTTCTGAC
**For the second bit:**<br><br>40, 41, 42, 43, 44, 45 and 46	AATATAATCCTGATTGTTTGGATTATACTTCTGAATAATGGAAGGG
	CACTAACAACTCCTCTTTTGAGGAACAAGTTTTCTTGTTAATAGATTA
	GAGCCGTCAATCCTCTTTTGAGGAACAAGTTTTCTTGTTAGATAATAC
	ATTTGAGGATTCCTCTTTTGAGGAACAAGTTTTCTTGTTTAGAAGTAT
	TAGACTTTACTCCTCTTTTGAGGAACAAGTTTTCTTGTAAACAATTCG
	ACAACTCGTATCCTCTTTTGAGGAACAAGTTTTCTTGTTTAAATCCTT
	TGCCCGAACGTCCTCTTTTGAGGAACAAGTTTTCTTGTTTATTAATTT
	TAAAAGTTTGTCCTCTTTTGAGGAACAAGTTTTCTTGTAGTAACATTA
	TCATTTTGCGTCCTCTTTTGAGGAACAAGTTTTCTTGTGAACAAAGAA
	ACCACCAGAATCCTCTTTTGAGGAACAAGTTTTCTTGTGGAGCGGAAT
	TATCATCATATCCTCTTTTGAGGAACAAGTTTTCTTGTTTCCTGATTA
	TCAGATGATGTCCTCTTTTGAGGAACAAGTTTTCTTGTGCAATTCATC
**For the third bit:**	TGTAAATGCTGATGCAAATCCAATCGCAAGACAAAGAACGCGAGAA

54, 55, 56, 57, 58, 59 and 60	TACCTTTTTTTCCTCTTTTGAGGAACAAGTTTTCTTGTAATGGAAACA
	GTACATAAATTCCTCTTTTGAGGAACAAGTTTTCTTGTCAATATATGT
	GAGTGAATAATCCTCTTTTGAGGAACAAGTTTTCTTGTCCTTGCTTCT
	GTAAATCGTCTCCTCTTTTGAGGAACAAGTTTTCTTGTGCTATTAATT
	AATTTTCCCTTCCTCTTTTGAGGAACAAGTTTTCTTGTTAGAATCCTT
	GAAAACATAGTCCTCTTTTGAGGAACAAGTTTTCTTGTCGATAGCTTA
	GATTAAGACGTCCTCTTTTGAGGAACAAGTTTTCTTGTCTGAGAAGAG
	TCAATAGTGATCCTCTTTTGAGGAACAAGTTTTCTTGTATTTATCAAA
	ATCATAGGTCTCCTCTTTTGAGGAACAAGTTTTCTTGTTGAGAGACTA
	CCTTTTTAACTCCTCTTTTGAGGAACAAGTTTTCTTGTCTCCGGCTTA
	GGTTGGGTTATCCTCTTTTGAGGAACAAGTTTTCTTGTTATAACTATA
**For the fourth bit:** 68, 69, 70, 71, 72, 73 and 74	TCATCGAGAACAAGCAAGCCGTTTTTATTTTCATCGTAGGAATCAT
	AGAATATAAATCCTCTTTTGAGGAACAAGTTTTCTTGTGTACCGACAA
	AAGGTAAAGTTCCTCTTTTGAGGAACAAGTTTTCTTGTAATTCTGTCC
	AGACGACGACTCCTCTTTTGAGGAACAAGTTTTCTTGTAATAAACAAC
	ATGTTCAGCTTCCTCTTTTGAGGAACAAGTTTTCTTGTAATGCAGAAC
	GCGCCTGTTTTCCTCTTTTGAGGAACAAGTTTTCTTGTATCAACAATA
	GATAAGTCCTTCCTCTTTTGAGGAACAAGTTTTCTTGTGAACAAGAAA
	AATAATATCCTCCTCTTTTGAGGAACAAGTTTTCTTGTCATCCTAATT
	TACGAGCATGTCCTCTTTTGAGGAACAAGTTTTCTTGTTAGAAACCAA
	TCAATAATCGTCCTCTTTTGAGGAACAAGTTTTCTTGTGCTGTCTTTC
	CTTATCATTCTCCTCTTTTGAGGAACAAGTTTTCTTGTCAAGAACGGG
	TATTAAACCATCCTCTTTTGAGGAACAAGTTTTCTTGTAGTACCGCAC
**For the fifth bit:** 82, 83, 84, 85, 86, 87 and 88	AGATAGCCGAACAAAGTTACCAGAAGGAAACCGAGGAAACGCAATA
	AAAAATGAAATCCTCTTTTGAGGAACAAGTTTTCTTGTATAGCAGCCT
	TTACAGAGAGTCCTCTTTTGAGGAACAAGTTTTCTTGTAATAACATAA
	AAACAGGGAATCCTCTTTTGAGGAACAAGTTTTCTTGTGCGCATTAGA
	CGGGAGAATTTCCTCTTTTGAGGAACAAGTTTTCTTGTAACTGAACAC
	CCTGAACAAATCCTCTTTTGAGGAACAAGTTTTCTTGTGTCAGAGGGT
	AATTGAGCGCTCCTCTTTTGAGGAACAAGTTTTCTTGTTAATATCAGA
	GAGATAACCCTCCTCTTTTGAGGAACAAGTTTTCTTGTACAAGAATTG

AGTTAAGCCCTCCTCTTTTGAGGAACAAGTTTTCTTGTAATAATAAGA
GCAAGAAACATCCTCTTTTGAGGAACAAGTTTTCTTGTATGAAATAGC
AATAGCTATCTCCTCTTTTGAGGAACAAGTTTTCTTGTTTACCGAAGC
CCTTTTTAAGTCCTCTTTTGAGGAACAAGTTTTCTTGTAAAAGTAAGC

Table S3

Table S3. Overhang sequences for DNA nanostructure with ´11111´ and one overhang as shown in Fig.4a. The sequences for overhangs are labelled as: Bold – crRNA target region, Red – Mutation, Blue – PAM).

Serovar_replaced oligonucleotides from Table S1.	Sequence (5′→3′)
Typhi_142	GATGGTTTAATTTCAACTTTAATCATTGTGAATTACCTTTCAAAGAGAA GCGACCTCGTGAGAGCAAGCGGACCTCATAAAGTGCGTCGT
Typhi_143	ACGACGCACTTTAT**GAGGTCCGCTTGCTCTCACGAGG**TCGCTTCTCTTT GTTTATGCGATTTTAAGAACTGGCTCATTATACCAGTCAGG
Newport_142	GATGGTTTAATTTCAACTTTAATCATTGTGAATTACCTTTCAAAGAGAA GCGACCTCGCGAGAGCAAGCGGACCTCATAAAGTGCGTCGT
Newport_143	ACGACGCACTTTAT**GAGGTCCGCTTGCTCTCGCGAGG**TCGCTTCTCTTT GTTTATGCGATTTTAAGAACTGGCTCATTATACCAGTCAGG

Table S4

Table S4. Oligonucleotides to assemble RNA ID 'C22' designed for serovar *Salmonella* Typhi. Lowercase portions are overhangs for biotinylated sequences to bind to and bold is the target area of the dCas9.

Oligonucleotide Number	Sequence (5′→3′)
1	AAGGAGGTGATCCAACCGCAGGTTCCCCTACGGTTACCTT
2	GTTACGACTTCACCCCAGTCATGAATCACAAAGTGGTAAG
3	CGCCCTCCCGAAGGTTAAGCTACCTACTTCTTTTGCAACC
4	CACTCCCATGGTGTGACGGGCGGTGTGTACAAGGCCCGGG
5	AACGTATTCACCGTGGCATTCTGATCCACGATTACTAGCG
6	ATTCCGACTTCATGGAGTCGAGTTGCAGACTCCAATCCGGACTAC
7	GACGCACTTTAT**GAGGTCCGCTTGCTCTCACG**AGGTCGCT
8	TCTCTTTGTATGCGCCATTGTAGCACGTGTGTAGC
9	CCTGGTCGTAAGGGCCATGATGACTTGACGTCATCCCCAC
10	CTTCCTCCAGTTTATCACTGGCAGTCTCCTTTGAGTTCCC
11	GGCCGGACCGCTGGCAACAAAGGATAAGGGTTGCGCTCGT
12	TGCGGGACTTAACCCAACATTTCACAACACGAGCTGACGA
13	CAGCCATGCAGCACCTGTCTCACAGTTCCCGAAGGCACCA
14	ATCCATCTCTGGAAAGTTCTGTGGATGTCAAGACCAGGTA
15	AGGTTCTTCGCGTTGCATCGAATTAAACCACATGCTCCAC
16	CGCTTGTGCGGGCCCCCGTCAATTCATTTGAGTTTTAACC
17	TTGCGGCCGTACTCCCCAGGCGGTCTACTTAACGCGTTAG
18	CTCCGGAAGCCACGCCTCAAGGGCACAACCTCCAAGTAGA
19	CATCGTTTACGGCGTGGACTACCAGGGTATCTAATCCTGT
20	TTGCTCCCCACGCTTTCGCACCTGAGCGTCAGTCTTTGTC
21	CAGGGGGCCGCCTTCGCCACCGGTATTCCTCCAGATCTCT
22	ACGCATTTCACCGCTACACCTGGAATTCTACCCCCCTCTA
23	CAAGACTCAAGCCTGCCAGTTTCGAATGCAGTTCCCAGGT
24	TGAGCCCGGGGATTTCACATCCGACTTGACAGACCGCCTG
25	CGTGCGCTTTACGCCCAGTAATTC
26	CGATTAACGCTTGCACCCTCCGTA
27	TTACCGCGGCTGCTGGCACGGAGTTAGCCGtttggatatcactcattagtggt
28	GTGCTTCTTCTGCGGGTAACGTCAATTGCTtttggatatcactcattagtggt
29	GCGGTTATTAACCACAACACCTTCCTCCCCGCTGAAAGTA
30	CTTTACAACCCGAAGGCCTTCTTCATACACGCGGCATGGC
31	TGCATCAGGCTTGCGCCCATTGTGCAATATTCCCCACTGC
32	TGCCTCCCGTAGGAGTCTGGACCGT
33	GTCTCAGTTCCAGTGTGGCTGGTCA
34	TCCTCTCAGACCAGCTAGGGATCGTCGCCTtttggatatcactcattagtggt

35	TGGTGAGCCGTTACCTCACCAACAAGCTAAtttggatatcactcattagtggt
36	TCCCATCTGGGCACATCTGATGGCAAGAGGCCCGAAGGTC
37	CCCCTCTTTGGTCTTGCGACGTTATGCGGTATTAGCCACC
38	GTTTCCAGTAGTTATCCCCCTCCATCAGGCAGTTTCCCAG
39	ACATTACTCACCCGTCCGCCACTCGTCAGCAAAGCAGCA
40	AGCTGCTTCCTGTTACCGTTCGACTTGCATGTGTTAGGCC
41	TGCCGCCAGCGTTCAATCTGAGCCATGATCAAACTCT

Table S5

Table S5. *E.coli* J01859.1 16S rRNA oligonucleotide sequences for ID `1131`.

Oligonucleotide Number	Sequence (5′→3′)
1	TAAGGAGGTGATCCAACCGCAGGTTCCCCTACGGTTAC
2	CTTGTTACGACTTCACCCCAGTCATGAATCACAAAGTG
3	GTAAGCGCCCTCCCGAAGGTTAAGCTACCTACTTCTTT
4	TGCAACCCACTCCCATGGTGTGACGGGCGGTGTGTACA
5	AGGCCCGGGAACGTATTCACCGTGGCATTCTGATCCAC
6	GATTACTAGCGATTCCGACTTCATGGAGTCGAGTTGCA
7	GACTCCAATCCGGACTACGACGCACTTTATGAGGTCCG
8	CTTGCTCTCGCGAGGTCGCTTCTCTTTGTATGCGCCAT
9	TGTAGCACGTGTGTAGCCCTGGTCGTAAGGGCCATGAT
10	GACTTGACGTCATCCCCACCTTCCTCCAGTTTATCACT
11	GGCAGTCTCCTTTGAGTT
12	CCCGGCCGGACCGCTGGCAACAAAGtttggatatcactcattagtggt
13	GATAAGGGTTGCGCTCGTTGCGGGACTTAACCCAACAT
14	TTCACAACACGAGCTGACGACAGCCATGCAGCACCTGT
15	CTCACGGTTCCCGAAGGCACATTCTCATCTCTGAAAAC
16	TTCCGTGGATGTCAAGACCAGGTAAGGTTCTTCGCGTT
17	GCATCGAATTAAACCACATGCTC
18	CACCGCTTGTGCGGGCCCCCGTCAAtttggatatcactcattagtggt
19	TTCATTTGAGTTTTAACCTTGCGGCCGTACTCCCCAGG
20	CGGTCGACTTAACGCGTTAGCTCCGGAAGCCACGCCTC
21	AAGGGCACAACCTCCAAGTCGACATCGTTTACGGCGTG
22	GACTACCAGGGTATCTAATCCTGTTTGCTCCCCACGCT
23	TTCGCACCTGAGCGTCAGTCTTC
24	GTCCAGGGGGCCGCCTTCGCCACCGtttggatatcactcattagtggt
25	GTATTCCTCCAGATCTCTACGCATTtttggatatcactcattagtggt
26	TCACCGCTACACCTGGAATTCTACCtttggatatcactcattagtggt
27	CCCCTCTACGAGACTCAAGCTTGCCAGTATCAGATGCA
28	GTTCCCAGGTTGAGCCCGGGGATTTCACATCTGACTTA
29	ACAAACCGCCTGCGTGCGCTTTACGCCCAGTAATTCCG
30	ATTAACGCTTGCACCCTCCGTATTAC
31	CGCGGCTGCTGGCACGGAGTTAGCC
32	GGTGCTTCTTCTGCGGGTAACGTCAtttggatatcactcattagtggt
33	ATGAGCAAAGGTATTAACTTTACTCCCTTCCTCCCCGC
34	TGAAAGTACTTTACAACCCGAAGGCCTTCTTCATACAC
35	GCGGCATGGCTGCATCAGGCTTGCGCCCATTGTGCAAT
36	ATTCCCCACTGCTGCCTCCCGTAGGAGTCTGGACCGTG

37	TCTCAGTTCCAGTGTGGCTGGTCATCCTCTCAGACCAG
38	CTAGGGATCGTCGCCTAGGTGAGCCGTTACCCCACCTA
39	CTAGCTAATCCCATCTGGGCACATCCGATGGCAAGAGG
40	CCCGAAGGTCCCCCTCTTTGGTCTTGCGACGTTATGCG
41	GTATTAGCTACCGTTTCCAGTAGTTATCCCCCTCCATC
42	AGGCAGTTTCCCAGACATTACTCACCCGTCCGCCACTC
43	GTCAGCAAAGAGCAAGCTTCTTCCTGTTACCGTTCGAC
44	TTGCATGTGTTAGGCCTGCCGCCAGCGTTCAATCTGAG
45	CCATGATCAAACTCTTCAATTT

Table S6

Table S6. *A. baumannii* 16S rRNA oligonucleotide sequences for ID `0331`.

Oligonucleotide Number	Sequence (5′→3′)
1	TAAGGAGGTGATCCAGCCGCAGGTTCCCCTACGGCTAC
2	CTTGTTACGACTTCACCCCAGTCATCGGCCACACCGTG
3	GTAACCGCCCTCTTTGCAGTTAGGCTAGCTACTTCTGG
4	TGCAACAAACTCCCATGGTGTGACGGGCGGTGTGTACA
5	AGGCCCGGGAACGTATTCACCGCGGCATTCTGATCCGC
6	GATTACTAGCGATTCCGACTTCATGGAGTCGAGTTGCA
7	GACTCCAATCCGGACTACGATCGGCTTTTTGAGATTAG
8	CATCACATCGCTGTGTAGCAACCCTTTGTACCGACCAT
9	TGTAGCACGTGTGTAGCCCTGGCCGTAAGGGCCATGAT
10	GACTTGACGTCGTCCCCGCCTTCCTCCAGTTTGTCACT
11	GGCAGTATCCTTAAAGTTCCCATCCGAAATGCTGGCAA
12	GTAAGGAAAAGGGTTGCGCTCGTTGCGGGACTTAACCC
13	AACATCTCACGACACGAGCTGACGACAGCCATGCAGCA
14	CCTGTATCTAGATTCCCGAAGGCACCAATCCATCTCTG
15	GAAAGTTTCTAGTATGTCAAGGCCAGGTAAGGTTCTTC
16	GCGTTGCATCGAATTAAACCACAT
17	GCTCCACCGCTTGTGCGGGCCCCCGtttggatatcactcattagtggt
18	TCAATTCATTTGAGTTTTAGTCTTGtttggatatcactcattagtggt
19	CGACCGTACTCCCCAGGCGGTCTACtttggatatcactcattagtggt
20	TTATCGCGTTAGCTGCGCCACTAAAGCCTCAAAGGCCC
21	CAACGGCTAGTAGACATCGTTTACGGCATGGACTACCA
22	GGGTATCTAATCCTGTTTGCTCCCCATGCTTTCGTACC
23	TCAGCGTCAGTATTAGGCCAGATGGCTGCCTTCGCCAT
24	CGGTATTCCTCCAGATCTCTACG
25	CATTTCACCGCTACACCTGGAATTCtttggatatcactcattagtggt
26	TACCATCCTCTCCCATACTCTAGCTtttggatatcactcattagtggt
27	CACCAGTATCGAATGCAATTCCCAAtttggatatcactcattagtggt
28	GTTAAGCTCGGGGATTTCACATCCGACTTAATAAGCCG
29	CCTACGCACGCTTTACGCCCAGTAAATCCGATTAACGC
30	TCGCACCCTCTGTATTACCGCGGCTGCTGGCACAGAGT
31	TAGCCGGTGCTTATTCTGCGAGTAACGTCCACTATCTC
32	TAGGTATTAACTAAAGTAGCCTC
33	CTCCTCGCTTAAAGTGCTTTACAACtttggatatcactcattagtggt
34	CATAAGGCCTTCTTCACACACGCGGCATGGCTGGATCA
35	GGGTTCCCCCCATTGTCCAATATTCCCCACTGCTGCCT
36	CCCGTAGGAGTCTGGGCCGTGTCTCAGTCCCAGTGTGG

37	CGGATCATCCTCTCAGACCCGCTACAGATCGTCGCCTT
38	GGTAGGCCTTTACCCCACCAACTAGCTAATCCGACTTA
39	GGCTCATCTATTAGCGCAAGGTCCGAAGATCCCCTGCT
40	TTCTCCCGTAGGACGTATGCGGTATTAGCATCCCTTTC
41	GAGATGTTGTCCCCCACTAATAGGCAGATTCCTAAGCA
42	TTACTCACCCGTCCGCCGCTAGGTCCAGTAGCAAGCTA
43	CCTTCCCCCGCTCGACTTGCATGTGTTAAGCCTGCCGC
44	CAGCGTTCAATCTGAGCCATGATCAAACTCTTCAGTTA

Table S7

Table S7. *A. baumannii* gp100 mRNA oligonucleotide sequences for ID `3113`.

Oligonucleotide Number	Sequence (5′→3′)
1	TTACGCATTATTAACTAAATTCCATATCTTATGAAAAT
2	CTGTATTTTGATTCCACCATAATACTTTAAAACCCATT
3	GCTTCATATTCTTTACCCAAAGTAGACTCATTACATAA
4	GCAAACGTTAGGATATGGAAATCTTTT
5	TCCTAAACATAATTCATCATTTTCAAC
6	TACACTTTTTATTTCTTCATTAATAtttggatatcactcattagtggt
7	TAAAGTGGATGAGGTCTAAAAAAAAtttggatatcactcattagtggt
8	CTTTAATCCCTTTATCTTTCAAGAAtttggatatcactcattagtggt
9	TTTATATAATAACAGTTCCTGATTTATAAAAAATGGAT
10	TACTAATTATCAATACTTCACATTTATCAGAAAAGTTG
11	ATATCTAAACTAATTTTCATATAATCAATTTTATTAGG
12	TATACCCTCTAATATATTTTTTAGAAAAATACTCAATT
13	GAGATTTATCAAAAACTTTTAAA
14	AAAGAAATATTTGGTATTTTATGCTtttggatatcactcattagtggt
15	TAACAACATAATTTTCTGTTAAAATTCCATGCTGTATT
16	ATCTCAATATCAAAATTACCAATATTTGACAAAACAGT
17	TAACCACCTATCATAATGATTTACCACACATATTTTAT
18	TTATATTTCTTTTATTTAATACAATAACAAATTCATAA
19	AATAAAATTAATTCAAAAATTGC
20	TGTTAAGTGTAAATAATATTTAATAtttggatatcactcattagtggt
21	TTAACAAAAAATATCATTTTAAAGTAAAGTATAATTGA
22	ATGAATAAAACTCTTCAGGATAACTAGAGGACTTAAAT
23	AGCCAACAAAACAATAAACTTTATTATTAATACATTGC
24	AAAAACTCTATATCAGAAGCATATTCTTCATAAATTGC
25	AATTACTTTTGGAGAGTTAGAAA
26	AAGATAAAAAATATTGATCATTTTCtttggatatcactcattagtggt
27	AAGATTTGATGTATTTCTACTCCTTtttggatatcactcattagtggt
28	CCAAAAAAAACAAAAAATTCAATAGtttggatatcactcattagtggt
29	CAAAATAAATTGGTATTAAAATAGAAGATAGCACAGAA
30	ATCCATAAAGGAATTTTATCTTTGAATCGTAGCATTTT
31	GAAAATAGATAGCTTTTTCGAGCCATAATTTAGCTTCT
32	CAAAAAGATAATTATCTACTGAAATTTCATTTTCAATA
33	GCTTTTTTTATAATTTCATA
34	TTTCTTATTTTTTAGGCACAT

Table S8

Table S8. Human 18S rRNA oligonucleotide sequences for ID `1111`.

Oligonucleotide Number	Sequence (5′→3′)
1	TAATGATCCTTCCGCAGGTTCACCTACGGAAACCTTGT
2	TACGACTTTTACTTCCTCTAGATAGTCAAGTTCGACCG
3	TCTTCTCAGCGCTCCGCCAGGGCCGTGGGCCGACCCCG
4	GCGGGGCCGATCCGAGGGCCTCACTAAACCATCCAATC
5	GGTAGTAGCGACGGGCGGTGTGTACAAAGGGCAGGGAC
6	TTAATCAACGCAAGCTTATGACCCGCACTTACTGGGAA
7	TTCCTCGTTCATGGGGAATAATTGC
8	AATCCCCGATCCCCATCACGAATGG
9	GGTTCAACGGTCCTCTTTTGAGGAACAAGTTTTCTTGTGTTACCCGCG
10	CCTGCCGGCGTCCTCTTTTGAGGAACAAGTTTTCTTGTTAGGGTAGGC
11	ACACGCTGAGTCCTCTTTTGAGGAACAAGTTTTCTTGTCCAGTCAGTG
12	TAGCGCGCGTTCCTCTTTTGAGGAACAAGTTTTCTTGTGCAGCCCCGG
13	ACATCTAAGGTCCTCTTTTGAGGAACAAGTTTTCTTGTGCATCACAGA
14	CCTGTTATTGTCCTCTTTTGAGGAACAAGTTTTCTTGTCTCAATCTCG
15	GGTGGCTGAACGCCACTTGTCCCTCTAAGAAGTTGGGG
16	GACGCCGACCGCTCGGGGGTCGCGTAACTAGTTAGCAT
17	GCCAGAGTCTCGTTCGTTATCGGAATTAACCAGACAAA
18	TCGCTCCACCAACTAAGAACGGCCATGCACCACCACCC
19	ACGGAATCGAGAAAGAGCTATCAATCTGTCAATCCTGT
20	CCGTGTCCGGGCCGGGTGAGGTTTCCCGTGTTGAGTCA
21	AATTAAGCCGCAGGCTCCACTCCTG
22	GTGGTGCCCTTCCGTCAATTCCTTT
23	AAGTTTCAGCTCCTCTTTTGAGGAACAAGTTTTCTTGTTTTGCAACCA
24	TACTCCCCCCTCCTCTTTTGAGGAACAAGTTTTCTTGTGGAACCCAAA
25	GACTTTGGTTTCCTCTTTTGAGGAACAAGTTTTCTTGTTCCCGGAAGC
26	TGCCCGGCGGTCCTCTTTTGAGGAACAAGTTTTCTTGTGTCATGGGAA
27	TAACGCCGCCTCCTCTTTTGAGGAACAAGTTTTCTTGTGCATCGCCGG
28	TCGGCATCGTTCCTCTTTTGAGGAACAAGTTTTCTTGTTTATGGTCGG
29	AACTACGACGGTATCTGATCGTCTTCGAACCTCCGACT
30	TTCGTTCTTGATTAATGAAAACATTCTTGGCAAATGCT
31	TTCGCTCTGGTCCGTCTTGCGCCGGTCCAAGAATTTCA
32	CCTCTAGCGGCGCAATACGAATGCCCCCGGCCGTCCCT
33	CTTAATCATGGCCTCAGTTCCGAAAACCAACAAAATAG
34	AACCGCGGTCCTATTCCATTATTCCTAGCTGCGGTATC
35	CAGGCGGCTCGGGCCTGCTTTGAAC
36	ACTCTAATTTTTTCAAAGTAAACGC

37	TTCGGGCCCCTCCTCTTTTGAGGAACAAGTTTTCTTGTGCGGGACACT
38	CAGCTAAGAGTCCTCTTTTGAGGAACAAGTTTTCTTGTCATCGAGGGG
39	GCGCCGAGAGTCCTCTTTTGAGGAACAAGTTTTCTTGTGCAAGGGGCG
40	GGGACGGGCGTCCTCTTTTGAGGAACAAGTTTTCTTGTGTGGCTCGCC
41	TCGCGGCGGATCCTCTTTTGAGGAACAAGTTTTCTTGTCCGCCCGCCC
42	GCTCCCAAGATCCTCTTTTGAGGAACAAGTTTTCTTGTTCCAACTACG
43	AGCTTTTTAACTGCAGCAACTTTAATATACGCTATTGG
44	AGCTGGAATTACCGCGGCTGCTGGCACCAGACTTGCCC
45	TCCAATGGATCCTCGTTAAAGGATTTAAAGTGGACTCA
46	TTCCAATTACAGGGCCTCGAAAGAGTCCTGTATTGTTA
47	TTTTTCGTCACTACCTCCCCGGGTCGGGAGTGGGTAAT
48	TTGCGCGCCTGCTGCCTTCCTTGGATGTGGTAGCCGTT
49	TCTCAGGCTCCCTCTCCGGAATCGA
50	ACCCTGATTCCCCGTCACCCGTGGT
51	CACCATGGTATCCTCTTTTGAGGAACAAGTTTTCTTGTGGCACGGCGA
52	CTACCATCGATCCTCTTTTGAGGAACAAGTTTTCTTGTAAGTTGATAG
53	GGCAGACGTTTCCTCTTTTGAGGAACAAGTTTTCTTGTCGAATGGGTC
54	GTCGCCGCCATCCTCTTTTGAGGAACAAGTTTTCTTGTCGGGGGGCGT
55	GCGATCGGCCTCCTCTTTTGAGGAACAAGTTTTCTTGTCGAGGTTATC
56	TAGAGTCACCTCCTCTTTTGAGGAACAAGTTTTCTTGTAAAGCCGCCG
57	GCGCCCGCCCCCCGGCCGGGGCCGGAGAGGGGCTGACC
58	GGGTTGGTTTTGATCTGATAAATGCACGCATCCCCCCC
59	GCGAAGGGGGTCAGCGCCCGTCGGCATGTATTAGCTCT
60	AGAATTACCACAGTTATCCAAGTGGGAGAGGAGCGAGC
61	GACCAAAGGAACCATAACTGATTTAATGAGCCATTCGC
62	AGTTTCACTGTACCGGCCGTGCGTACTTAGACATGCAT
63	GGCTTAATCTTTGAGACAAGCATAT
64	GCTACTGGCAGGATCAACCAGGTA

Table S9

Table S9. Mice NR_003278.3 18S rRNA oligonucleotide sequences for ID `3232`.

Oligonucleotide Number	Sequence (5′→3′)
1	TTAATGATCCTTCCGCAGGTTCACCTACGGAAACCTTG
2	TTACGACTTTTACTTCCTCTAGATAGTCAAGTTCGACC
3	GTCTTCTCAGCGCTCCGCCAGGGCCGTGGGCCGACCCC
4	GGCGGGGCCGATCCGAGGGCCTCACTAAACCATCCAAT
5	CGGTAGTAGCGACGGGCGGTGTGTACAAAGGGCAGGGA
6	CTTAATCAACGCAAGCTTATGACCCGCACTTACTGGGA
7	ATTCCTCGTTCATGGGGAATAATTGCAATCCCCGATCC
8	CCATCACGAATGGGGTTCAACGGGTTACCCGCGCCTGC
9	CGGCGCAGGGTAGGCACACGCTGAGCCAGTCAGTGTAG
10	CGCGCGTGCAGCCCCGGACATCTAAGGGCATCACAGAC
11	CTGTTATTGCTCAATCTCGGGTGGCTGAACGCCACTTG
12	TCCCTCTAAGAAGTTGGGGGACGCCGACCGCTCGGGGG
13	TCGCGTAACTAGTTAGCATGCCAGAGTCTCGTTCGTTA
14	TCGGAATTAACCAGACAAAT
15	CGCTCCACCAACTAAGAACGG
16	CCATGCACCACCACCCACGGAATCGtttggatatcactcattagtggt
17	AGAAAGAGCTATCAATCTGTCAATCtttggatatcactcattagtggt
18	CTGTCCGTGTCCGGGCCGGGTGAGGTTTCCCGTGTTGA
19	GTCAAATTAAGCCGCAGGCCCACTCCTGGTGGTGCCCT
20	TCCGTCAATTCCTTTAAGTTTCAGCTTTGCAACCATAC
21	TCCCCCCGGAACCCAAAGACTTTGGTTTCCCGGAAGCT
22	GCCCGGCGGGTCATGGGAATAAC
23	GCCGCCGCATCGCCAGTCGGCATCGtttggatatcactcattagtggt
24	TTTATGGTCGGAACTACGACGGTATtttggatatcactcattagtggt
25	CTGATCGTCTTCGAACCTCCGACTTtttggatatcactcattagtggt
26	TCGTTCTTGATTAATGAAAACATTCTTGGCAAATGCTT
27	TCGCTCTGGTCCGTCTTGCGCCGGTCCAAGAATTTCAC
28	CTCTAGCGGCGCAATACGAATGCCCCCGGCCGTCCCTC
29	TTAATCATGGCCTCAGTTCCGAAAACCAACAAAATAGA
30	ACCGCGGTCCTATTCCATTATTC
31	CTAGCTGCGGTATCCAGGCGGCTCGtttggatatcactcattagtggt
32	GGCCTGCTTTGAACACTCTAATTTTtttggatatcactcattagtggt
33	TTCAAAGTAAACGCTTCGGGCCCCGCGGGACACTCAGC
34	TAAGAGCATCGAGGGGGCGCCGAGAGGCAAGGGGCGGG
35	GACGGGCGGTGACTCGCCTCGCGGCGGACCGCCCGCCC
36	GCTCCCAAGATCCAACTACGAGCTTTTTAACTGCAGCA

37	ACTTTAATATACGCTATTGGAGC
38	TGGAATTACCGCGGCTGCTGGCACCtttggatatcactcattagtggt
39	AGACTTGCCCTCCAATGGATCCTCGtttggatatcactcattagtggt
40	TTAAAGGATTTAAAGTGGACTCATTtttggatatcactcattagtggt
41	CCAATTACAGGGCCTCGAAAGAGTCCTGTATTGTTATT
42	TTTCGTCACTACCTCCCCGGGTCGGGAGTGGGTAATTT
43	GCGCGCCTGCTGCCTTCCTTGGATGTGGTAGCCGTTTC
44	TCAGGCTCCCTCTCCGGAATCGAACCCTGATTCCCCGT
45	CACCCGTGGTCACCATGGTAGGCACGGCGACTACCATC
46	GAAAGTTGATAGGGCAGACGTTCGAATGGGTCGTCGCC
47	GCCACGGGGGGCGTGCGATCGGCCCGAGGTTATCTAGA
48	GTCACCAAGCCGCCGGCGCCCGACCCCCGGCCGGAGCC
49	GGGAGGGAGCTCACCGGGTTGGTTTTGATCTGATAAAT
50	GCACGCATCCCCCCCCGGGAAGGGGGGTCAGCGCCCGT
51	CGGCATGTATTAGCTCTAGAATTACCACAGTTATCCAA
52	GTAGGAGAGGAGCGAGCGACCAAAGGAACCATAACTGA
53	TTTAATGAGCCATTCGCAGTTTCACTGTACCGGCCGTG
54	CGTACTTAGACATGCATGGCTTAATCTTTGAGACAAGC
55	ATATGCTACCTGGCAGGATCAACCAGGT

Table S10

Table S10. *Salmonella* Typhimurium 16S rRNA oligonucleotide sequences for ID `3113`.

Oligonucleotide Number	Sequence (5′→3′)
1	TAAGGAGGTGATCCAACCGCAGGTTNCCCTACGGTTAC
2	CTTGTTACGACTTCACCCCAGTCATGAATCACAAAGTG
3	GTAAGCGCCCTCCCGAAGGTTAAGCTACCTACTTCTTT
4	TGCAACCCACTCCCATGGTGTGACGGGCGGTGTGTACA
5	AGGCCCGGGAACGTATTCACCGTGGCATTCTGATCCAC
6	GATTACTAGCGATTCCGACTTCATGGAGTCGAGTTGCA
7	GACTCCAATCCGGACTACGACGCACTTTATGAGGTCCG
8	CTTGCTCTCGCGAGGTCGCTTCTCTTTGTATGCGCCAT
9	TGTAGCACGTGTGTAGCCCTGGTCGTAAGGGCCATGAT
10	GACTTGACGTCATCCCCACCTTCCTC
11	CAGTTTATCACTGGCAGTCTCCTTTGA
12	GTTCCCGACCTAATCGCTGGCAACAtttggatatcactcattagtggt
13	AAGGATAAGGGTTGCGCTCGTTGCGtttggatatcactcattagtggt
14	GGACTTAACCCAACATTTCACAACAtttggatatcactcattagtggt
15	CGAGCTGACGACAGCCATGCAGCACCTGTCTCACAGTT
16	CCCGAAGGCACCAATCCATCTCTGGATTCTTCTGTGGA
17	TGTCAAGACCAGGTAAGGTTCTTCGCGTTGCATCGAAT
18	TAAACCACATGCTCCACCGCTTGTGCGGCCCCCGTCAA
19	TTCATTTGAGTTTTAACCTTGCG
20	GCCGTACTCCCCAGGCGGTCTACTTtttggatatcactcattagtggt
21	AACGCGTTACGTCCGGAAGCCACGCCTCAAGGGCACAA
22	CCTCCAAGTAGACATCGTTTACGGCGTGGACTACCAGG
23	GTATCTAATCCTGTTTGCTCCCCACGCTTTCGCACCTG
24	AGCGTCAGTCTTTGTCCAGGGGGCCGCCTTCGCCACCG
25	GTATTCCTCCAGATCTCTACGCA
26	TTTCACCGCTACACCTGGAATTCTAtttggatatcactcattagtggt
27	CCCCCCTCTACAAGACTCAAGCCTGCCAGTTTCGAATG
28	CAGTTCCCAGGTTGAGCCCGGGGATTTCACATCCGACT
29	TGACAGACCGCCTGCGTGCGCTTTACGCCCAGTAATTC
30	CGATTAACGCTTGCACCCTCCGTATTACCGCGGCTGCT
31	GGCACGGAGTTAGCCGGTGCTTC
32	TTCTGCGGGTAACGTCAATTGCTGCtttggatatcactcattagtggt
33	GGTTATTAACCACAACACCTTCCTCtttggatatcactcattagtggt
34	CCCGCTGAAAGTACTTTACAACCCGtttggatatcactcattagtggt
35	AAGGCCTTCTTCATACACGCGGCATGGCTGCATCAGGC
36	TTGCGCCCATTGTGCAATATTCCCCACTGCTGCCTCCC

37	GTAGGAGTCTGGACCGTGTCTCAGTTCCAGTGTGGCTG
38	GTCATCCTCTCAGACCAGCTAGGGATCGTCGCCTTGGT
39	GAGCCGTTACCTCACCAACTAGCTAATCCCATCTGGGC
40	ACATCTGATGGCAAGAGGCCCGAAGGTCCCCCTCTTTG
41	GTCTTGCGACGTTATGCGGTATTAGCCACCGTTTCCAG
42	TAGTTATCCCCCTCCATCAGGCAGTTTCCCAGACATTA
43	CTCACCCGTCCGCCACTNNNNAGCGAAGNGCAAGCTGC
44	TTCCTGTTACCGTTCGACTTGCATGTGTTAGGCCTGCC
45	GCCAGCGTTCAATCTGAGC
46	CATGATCAAACTCTTCAATT

Table S11

Table S11. MS2 rRNA oligonucleotide sequences for ID `111`.

Oligonucleotide Number	Sequence (5′→3′)
1	CACTCCGTTCCCTACAACGAGCCTAAATTCATATGACT
2	CGTTATAGCGGACCGCGTGTCTGATCCACGGCGCACAT
3	TGGTCTCGGACCAATAGAGCCGCTCTCAGAGCGCGGGG
4	GGTAACGGTTGCTTGTTCAGCGAACTTCTTGTAAGGCG
5	CTGCATCCTGCAACTTGTGCCCCATAGGAGCACCGTTG
6	GAGAACGTGCATTGCCCAAACAACGACGATCGGTAGCC
7	AGAGAGGAGGTTGCCAATAAGGCTACGGATGCTGGTTT
8	GTAAAACATCCGGATCCCATGACAAGGATTTGTCATGT
9	AAGAAACCTTCTCTATTTATCTGACCGCGATCACCATT
10	CGCCTCCCGTTCCTCTTTTGAGGAACAAGTTTTCTTGTAGCTTAGCGA
11	TAGCTAAGGTTCCTCTTTTGAGGAACAAGTTTTCTTGTACGACGGGTC
12	GCCTCGTCATTCCTCTTTTGAGGAACAAGTTTTCTTGTTACCAGAACC
13	TAAGGTCGGATCCTCTTTTGAGGAACAAGTTTTCTTGTTGCTTTGTGA
14	GCAATTCGTCTCCTCTTTTGAGGAACAAGTTTTCTTGTCCTTAAGTAA
15	GCAATTGCTGTCCTCTTTTGAGGAACAAGTTTTCTTGTTAAAGTCGTC
16	ACTGTGCGGATCACCGCTTCCAGTAGCGACAG
17	AAGCAATTGATTGGTAAATTTCGAGAGAAAGATCGCGA
18	GGAAGATCAATACATAAAGAGTTGAACTTCTTTGTTGT
19	CTTCGACATGGGTAATCCTCATGTTTGAATGGCCGGCG
20	TCTATTAGTAGATGCCGGAGTTTGCTGCGATTGCTGAG
21	GGAATCGGGTTTCCATCTTTTAGGAGACCTTGCATTGC
22	CTTAACAATAAGCTCGCAGTCGGAATTCGTAGCGAAAA
23	TTGGAATGGTTAGTTCCATATTTAAGTACGAACGCCAT
24	GCGGCTACAGGAAGCTCTACACCACCAACAGTCTGGGT
25	TGCCACTTTAGGCACCTCGACTTTGATGGTGTATTTGC
26	GATTCTGCGCAGAGCTCTGACGAACGCTACAGGTTACT
27	TTGTAAGCCTGTGAACGCGAGTTAGAGCTGATCCATTC
28	AGCGACCCCGTTAGCGAAGTTGCTTGGGGCGACAGTCA
29	CGTCGCCAGTTCCTCTTTTGAGGAACAAGTTTTCTTGTTCCGCCATTG
30	TCGACGAGAATCCTCTTTTGAGGAACAAGTTTTCTTGTCGAACTGAGT
31	AAAGTTAGAATCCTCTTTTGAGGAACAAGTTTTCTTGTGCCATGCTTC
32	AAACTCCGGTTCCTCTTTTGAGGAACAAGTTTTCTTGTTGAGGGCTCT
33	ATCTAGAGAGTCCTCTTTTGAGGAACAAGTTTTCTTGTCCGTTGCCTG
34	ATTAATGCTATCCTCTTTTGAGGAACAAGTTTTCTTGTACGCATCTAA
35	GGTATGGACCATCGAGAAAGGAGACTTTACGT
36	ACGCGCCAGTTGTTGGCCATACGGATTGTACCCCTCGA

37	TGCATGGCTGAGATTTGGGCCTTAGCAGTGCCCTGTCT
38	CTCCACAGTCCACCCGTAGGGAGCGTCAACGCTTATGA
39	TGGACTCACCCGTTATTACGTCAGTAACTGTTCCTGAC
40	ATGTAGGAGCATCCCACGGGGGCCGTAAGGCCCTCGAG
41	CATGTTACCTACAGGTAGGAGCCAGTCGACAACGAATG
42	AGAAAGGCACCTTTTCCCACACTATACCTAGTGGGTTC
43	AAGATACCTAGAGACGACAACCATGCCAAACGTGCATC
44	GTTTATGTAAAACCATATCACGATACGTCGCGATATGT
45	TGCACGTTGTTCCTCTTTTGAGGAACAAGTTTTCTTGTCTGGAAGTTT
46	GCAGCTGGATTCCTCTTTTGAGGAACAAGTTTTCTTGTACGACAGACG
47	GCCATCTAACTCCTCTTTTGAGGAACAAGTTTTCTTGTTTGATGTTAG
48	TACCGACCTGTCCTCTTTTGAGGAACAAGTTTTCTTGTACGTACGGCT
49	CTCATAGGAATCCTCTTTTGAGGAACAAGTTTTCTTGTGAAACTCTTG
50	AAGGTGAACCTCCTCTTTTGAGGAACAAGTTTTCTTGTTTCGTAAGCA
51	TCTCATATGCACCCTGGATATCACTCATTAGT
52	GGTAACCAACCGAACTGCAACTCCAACCACCTGCCGGC
53	CACGTGTTTTGATCGAAACTTTCGATCTTCGTTTAGGG
54	CAAGGTAGCGGAGCGCCTGGCGCCAATTACCGCGACGA
55	GCGGCAGTGTACGCCTTCACGAGCGCAATGGTTTGCGT
56	CGCGAGTTGTGAGGCTGTCGACCTGGCCTCTGCTAAAG
57	CAACACCAAGGTTAAAATTACCCTGGGTGACCTTTTGC
58	AGGACTTCGGTCGACGCCCGGTTCGCAACGTTCTGCGG
59	CACTTCGATGTAAGTCAAGTTTTGGCTTACAGGGAAGA
60	GGCTGTAGCAGGAGCGTGCGTCGAGGGAGAAGCCGAAA
61	CCGGCTTTCTCCTCGTACGGGCGACCCCACGATGACCCACTTCGCTTGTAG
62	GCACCTTGATCTATCGATGTGACACTTAACGCCCCCCGTGAATACGGAGA
63	GGGGTAGTGCCACTGTTTCGTTTTGGCCCCAGTCGAGTTAAAACGACCGG
64	GAGTCCAGTTCGAACGATATTTTAAAGAGAATGAGTTATCTTCAGTCTCA
65	CCGTCCGCGTAAACGCGAACGGAGGGGACGAAGGTCTCGTTCTCCCTATC
66	AAGGGTACTAAAAGCTCGCACAGGTCAAACCTCCTAGGAATGGAATTCCG
67	GCTACCTACAGCGATAGCCATGGTAGCGTCTCGCTAAAGACATTAAAAAT
68	GGCATTAGCTCGACAGGAAGTTGAGCAGGACCCCGAAAGGGGTCCCACCC
69	TGGGTGGTAACTAGCCAAGCAGCTAGTTACCAAATCGGGAGAATCCCGGG
70	TCCTCTCTTTAGGGGGAGGTCCCTGGGCCGAAGCCCGCCCACCTTTCGGT
71	GGAGCCGGACCGCTTTCGCACCCGTGCTCTTTCGAGCACACCCACCCCGT
72	TTACGGGGGTCCCTCGGTCAGCTACCGAGGAGAGCTCGCTGGCCCACACT
73	CCTGAGGGAATGTGGGAACCGGCGTTAGCCACTCCGAAGTGCGTATAACG
74	CGCACGCCGGCGGACTTCATGCTGTCGGTGATTTCACCTCCAGTATGGAA
75	CCACGCTATGTAGCGACCACTGTCGTGCTTTTCGCTGAAGAACTTGCGTT
76	CTCGAGCGATACGAGCAAGACGGAAACCCGAGGTACGGGTATCCGCGAGC
77	AGCCGCCCGTACGGAGTCTTGGTGTATACCGAGACTGCCGTAGGCGGGCT

78	GACTACGTAGTAGTCGGCAGCGAGGTCCGTCCCACCGAAGAACATCGAAG
79	GCACCTGGGAGGAGAGCCGTACCCACACCTTATAGAGGCGTGGATCTGAC
80	ATACCTCCGACAACTCCCCAACCCCGTAGCCGATTTAATATCAGCATCAG
81	GGCGAAGAGATTGTCAACAGGTTTCTTGATGTAAAACGGTTTGACATCGA
82	CACCACGGTAAAAGTGCGCGCCGCAGCTCTCGCGAAAGAGCCCGGACACG
83	AACGTTTTACGAAGATTCGGTTTAAAACCGTAGTAGGCAAGTGCCTCTAG
84	CACACGGGGTGCAATCTCACTGGGACATATAATATCGTCCCCGTAGATGC
85	CTATGGTTCCGGCGTTACCAAAATGGATTTGGGTCGCTTTGACTATTGCC
86	CAGAATATCATGGACTCTAGCTCAAATGTGAACCCATTTCCCATTGTGGA
87	AAATAGTTCCCATCGTATCGTCTCGCCATCTACGATTCCGTAGTGTGAGC
88	GGATACGATCGAGATATGAATATAGCTCTGGTGGGAGAAAACTCCACACC
89	AGGCGATCGGAGATGGAATCGGATGCAGACGATAAGTCTATCGTCGCAAG
90	CGAACCATCTACGCTGCCCTGCTGAGCCAGACGCTGGTTGATCGATTGAT
91	CATTCAGGTCTATACCAACGGATTTGAGCCGGCGTCTGATGAAAGCACCG
92	ACCCCTTTCTGGAGGTACATATTCATATCAGGCTCCTTAC
93	AGGCAGCCCGATCTATTTTATTATTCTTCGGAACTGTAAA

Table S12

Table S12. M13 DNA oligonucleotide sequences for ID `111111`.

Replaced oligonucleotides from Table S1.	Sequence (5′→3′)
**For the first bit:** 26-30	ACATCACTTGTCCTCTTTTGAGGAACAAGTTTTCTTGTCCTGAGTAGA
	AGAACTCAAATCCTCTTTTGAGGAACAAGTTTTCTTGTCTATCGGCCT
	TGCTGGTAATTCCTCTTTTGAGGAACAAGTTTTCTTGTATCCAGAACA
	ATATTACCGCTCCTCTTTTGAGGAACAAGTTTTCTTGTCAGCCATTGC
	AACAGGAAAATCCTCTTTTGAGGAACAAGTTTTCTTGTACGCTCATGG
	AAATACCTACTCCTCTTTTGAGGAACAAGTTTTCTTGTATTTTGACGC
	TCAATCGTCTTCCTCTTTTGAGGAACAAGTTTTCTTGTGAAATGGATT
	ATTTACATTGTCCTCTTTTGAGGAACAAGTTTTCTTGTGCAGATTCAC
	CAGTCACACGACCAGTAATAAAAGGGACAT
**For the second bit:** 52-57	TTACCTGAGCAAAAGAAGATGATGAAACAAACATCAAGAAAACA
	AAATTAATTATCCTCTTTTGAGGAACAAGTTTTCTTGTCATTTAACAA
	TTTCATTTGATCCTCTTTTGAGGAACAAGTTTTCTTGTATTACCTTTT
	TTAATGGAAATCCTCTTTTGAGGAACAAGTTTTCTTGTCAGTACATAA
	ATCAATATATTCCTCTTTTGAGGAACAAGTTTTCTTGTGTGAGTGAAT
	AACCTTGCTTTCCTCTTTTGAGGAACAAGTTTTCTTGTCTGTAAATCG
	TCGCTATTAATCCTCTTTTGAGGAACAAGTTTTCTTGTTTAATTTTCC
	CTTAGAATCCTCCTCTTTTGAGGAACAAGTTTTCTTGTTTGAAAACAT
	AGCGATAGCTTCCTCTTTTGAGGAACAAGTTTTCTTGTTAGATTAAGA
	CGCTGAGAAGAGTCAATAGTGAAT
**For the third bit:** 79-85	TGAATCTTACCAACGCTAACGAGCGTCTTTCCAGAGCCTAATTTGCCAGT
	TACAAAATAATCCTCTTTTGAGGAACAAGTTTTCTTGTACAGCCATAT
	TATTTATCCCTCCTCTTTTGAGGAACAAGTTTTCTTGTAATCCAAATA
	AGAAACGATTTCCTCTTTTGAGGAACAAGTTTTCTTGTTTTTGTTTAA
	CGTCAAAAATTCCTCTTTTGAGGAACAAGTTTTCTTGTGAAAATAGCA
	GCCTTTACAGTCCTCTTTTGAGGAACAAGTTTTCTTGTAGAGAATAAC
	ATAAAAACAGTCCTCTTTTGAGGAACAAGTTTTCTTGTGGAAGCGCAT

	TAGACGGGAGTCCTCTTTTGAGGAACAAGTTTTCTTGTAATTAACTGA
	ACACCCTGAATCCTCTTTTGAGGAACAAGTTTTCTTGTCAAAGTCAGA
	GGGTAATTGAGCGCTAATATCAGAGAGATAACCCACAAGAATTGAGTTAAGCCCAA
**For the fourth bit:**	TCACAAACAAATAAATCCTCATTAAAGCCAGAATGGAAAGCGCAGTCTCTGAATTT
106-112	ACCGTTCCAGTCCTCTTTTGAGGAACAAGTTTTCTTGTTAAGCGTCAT
	ACATGGCTTTTCCTCTTTTGAGGAACAAGTTTTCTTGTTGATGATACA
	GGAGTGTACTTCCTCTTTTGAGGAACAAGTTTTCTTGTGGTAATAAGT
	TTTAACGGGGTCCTCTTTTGAGGAACAAGTTTTCTTGTTCAGTGCCTT
	GAGTAACAGTTCCTCTTTTGAGGAACAAGTTTTCTTGTGCCCGTATAA
	ACAGTTAATGTCCTCTTTTGAGGAACAAGTTTTCTTGTCCCCCTGCCT
	ATTTCGGAACTCCTCTTTTGAGGAACAAGTTTTCTTGTCTATTATTCT
	GAAACATGAATCCTCTTTTGAGGAACAAGTTTTCTTGTAGTATTAAGA
	GGCTGAGACTCCTCAAGAGAAGGATTAGGATTAGCGGGGTTTTGCTCAGT
**For the fifth bit:**	CAAAGTACAACGGAGATTTGTATC
134-139	ATCGCCTGATTCCTCTTTTGAGGAACAAGTTTTCTTGTAAATTGTGTC
	GAAATCCGCGTCCTCTTTTGAGGAACAAGTTTTCTTGTACCTGCTCCA
	TGTTACTTAGTCCTCTTTTGAGGAACAAGTTTTCTTGTCCGGAACGAG
	GCGCAGACGGTCCTCTTTTGAGGAACAAGTTTTCTTGTTCAATCATAA
	GGGAACCGAATCCTCTTTTGAGGAACAAGTTTTCTTGTCTGACCAACT
	TTGAAAGAGGTCCTCTTTTGAGGAACAAGTTTTCTTGTACAGATGAAC
	GGTGTACAGATCCTCTTTTGAGGAACAAGTTTTCTTGTCCAGGCGCAT
	AGGCTGGCTGTCCTCTTTTGAGGAACAAGTTTTCTTGTACCTTCATCA
	AGAGTAATCTTGACAAGAACCGGATATTCATTACCCAAATCAAC
**For the sixth bit:**	ACAGTTGATTCCCAATTCTGCGAACGAGTA
161-165	GATTTAGTTTTCCTCTTTTGAGGAACAAGTTTTCTTGTGACCATTAGA
	TACATTTCGCTCCTCTTTTGAGGAACAAGTTTTCTTGTAAATGGTCAA
	TAACCTGTTTTCCTCTTTTGAGGAACAAGTTTTCTTGTAGCTATATTT
	TCATTTGGGGTCCTCTTTTGAGGAACAAGTTTTCTTGTCGCGAGCTGA
	AAAGGTGGCATCCTCTTTTGAGGAACAAGTTTTCTTGTTCAATTCTAC
	TAATAGTAGTTCCTCTTTTGAGGAACAAGTTTTCTTGTAGCATTAACA
	TCCAATAAATTCCTCTTTTGAGGAACAAGTTTTCTTGTCATACAGGCA

AGGCAAAGAATCCTCTTTTGAGGAACAAGTTTTCTTGTTTAGCAAAAT

Table S13

Table S13. *Salmonella* Typhimurium 23S rRNA oligonucleotide sequences for ID `3113`.

Oligonucleotide Number	Sequence (5′→3′)
1	TTAAGCCTCACGGTTCATTAGTACCGGTTAGCTCAACG
2	CATCGCTGCGCTTACACACCCGGCCTATCAACGTCGTC
3	GTCTTCAACGTTCCTTCAGGAGACCTAAAGTCTCAGGG
4	AGAACTCATCTCGGGGCAAGTTTCGTGCTTAGATGCTT
5	TCAGCACTTATCTCTTCCGCATTTAGCTACCGGGCAGT
6	GCCATTGGCATGACAACCCGAACACCAGTGATGCGTCC
7	ACTCCGGTCCTCTCGTACTAGGAGCAGCCCCCCTCAGT
8	TCTCCAGCGCCCACGGCAGATAGGGACCGAACTGTCTC
9	ACGACGTTCTAAACCCAGCTCGCGTACCACTTTAAATG
10	GCGAACAGCCATACCCTTGGGACCTACTTCAGCCCCAG
11	GATGTGATGAGCCGACATCGAGGTGCCAAACACCGCCG
12	TCGATATGAACTCTTGGGCGGTATCAGCCTGTTATCCC
13	CGGAGTACCTTTTATCCGTTGAGCGATGGCCCTTCCAT
14	TCAGAACCACCGGATCACTATGACCTGCTTTCGCACCT
15	GCTCGCGCCGTCACGCTCGCAGTCAAGCTGGCTTATGC
16	CATTGCACTAACCTCCTGATGTCCGACCAGGATTAGCC
17	AACCTTCGTGCTCCTCCGTTACTCTTTAGGAGGAGACC
18	GCCCCAGTCAAACTACCCACCAGACACTGTCCGCAACC
19	CGGATTACGGGTCCACGTTAGAACATCAAACATTAAAG
20	GGTGGTATTTCAAGGTCGGCTCCATGCAGACTGGCGTC
21	CACACTTCAAAGCCTCCCACCTATCCTACACATCAAGG
22	CTCAATGTTCAGTGTCAAGCTATAGTAAAGGTTCACGG
23	GGTCTTTCCGTCTTGCCGCGGGTACACTGCATCTTCAC
24	AGCGAGTTCAATTTCACTGAGTCTCGGGTGGAGACAGC
25	CTGGCCATCATTACGCCATTCGTGCAGGTCGGAACTTA

26	CCCGACAAGGAATTTCGCTACCTTAGGACCGTTATAGT
27	TACGGCCGCCGTTTACCGGGGCTTCGATCAGGAGCTTC
28	GCTTGCGCTGACCCCATCAATTAACCTTCCGGCACCGG
29	GCAGGCGTCACACCGTATACGTCCACTTTCGT
30	GTTTGCACAGTGCTGTGTTTTTAATtttggatatcactcattagtggt
31	AAACAGTTGCAGCCAGCTGGTATCTtttggatatcactcattagtggt
32	TCGACTGACTTCAGCTCCGTGAGTAtttggatatcactcattagtggt
33	AATCACTTCACCTACGTGTCAGCGTGCCTTCTCCCGAA
34	GTTACGGCACCATTTTGCCTAGTTCCTTCACCCGAGTT
35	CTCTCAAGCGCCTTGGTATTCTCTACCTGACCACCTGT
36	GTCGGTTTGGGGTACGATTTGATGTTACCTGATGCTTA
37	GAGGCTTTTCCTGGAAGCAGGGC
38	ATTTGTTGCTTCAGCACCGTAGTGCtttggatatcactcattagtggt
39	CTCGTCATCACGCCTCAGTGTTAAAGTGAACCGGATTT
40	ACCTGGAACACACACCTACACGCTTAAACCGGGACAAC
41	CGTCGCCCGGCCAACATAGCCTTCTCCGTCCCCCCTTC
42	GCAGTAACACCAAGTACGGGAATATTAACCCGTTTCCC
43	ATCGACTACGCCTTTCGGCCTCG
44	CCTTAGGGGTCGACTCACCCTGCCCtttggatatcactcattagtggt
45	CGATTAACGTTGGACAGGAACCCTTGGTCTTCCGGCGA
46	GCGGGCTTTTCACCCGCTTTATCGTTACTTATGTCAGC
47	ATTCGCACTTCTGATACCTCCAGCAACCCTCACAGGTC
48	ACCTTCGCAGGCTTACAGAACGCTCCCCTACCCAACAA
49	CGCATCAGCGTCGCTGCCGCAGC
50	TTCGGTGCATGGTTTAGCCCCGTTAtttggatatcactcattagtggt
51	CATCTTCCGCGCAGGCCGACTCGACtttggatatcactcattagtggt
52	CAGTGAGCTATTACGCTTTCTTTAAtttggatatcactcattagtggt
53	ATGATGGCTGCTTCTAAGCCAACATCCTGGCTGTCTGG
54	GCCTTCCCACATCGTTTCCCACTTAACCATGACTTTGG

55	GACCTTAGCTGGCGGTCTGGGTTGTTTCCCTCTTCACG
56	ACGGACGTTAGCACCCGCCGTGTGTCTCCCGTGATAAC
57	ATTCTCCGGTATTCGCAGTTTGCATCGGGTTGGTAAGC
58	CGGGATGGCCCCCTAGCCGAAACAGTGCTCTACCCCCG
59	GAGATGAATTCACGAGGCGCTACCTAAATAGCTTTCGG
60	GGAGAACCAGCTATCTCCCGGTTTGATTGGCCTTTCAC
61	CCCCAGCCACAGGTCATCCGCTAATTTTTCAACATTAG
62	TCGGTTCGGTCCTCCAGTTAGTGTTACCCAACCTTCAA
63	CCTGCCCATGGCTAGATCACCGGGTTTCGGGTCTATAC
64	CCTGCAACTTAACGCCCAATTAAGACTCGGTTTCCCTC
65	CGGCTCCCCTATTCGGTTAACCTTGCTACAGAATATAA
66	GTCGCTGACCCATTATACAAAAGGTACGCAGTCACCCC
67	ACCCCAAAGCATTCACCGCTTGTTTTGTGTGTTGAATG
68	CTTTGGTGGTGGGGCTCCCACTGCTTGTACGTACACGG
69	TTTCAGGTTCTTTTTCACTCCCCTCGCCGGGGTTCTTT
70	TCGCCTTTCCCTCACGGTACTGGTTCACTATCGGTCAG
71	TCAGGAGTATTTAGCCTTGGAGGATGGTCCCCCCATAT
72	TCAGACAGGATACCACGTGTCCCGCCCTACTCATCGAG
73	CTCACAGCACATGCGCTTTTGTGTACGGGGCTGTCACC
74	CTGTATCGCGCGCCTTTCCAGACGCTTCCACTAACACA
75	CATGCTGATTCAGGCTCTGGGCTCCTCCCCGTTCGCTC
76	GCCGCTACTGGGGGAATCTCGGTTGATTTCTTTTCCTC
77	GGGGTACTTAGATGTTTCAGTTCCCCCGGTTCGCCTCA
78	TTAACCTATGGATTCAGTTAATGATAGTGTGACGAGTC
79	ACACTGGGTTTCCCCATTCGGGTATCGCCGGTTATAAC
80	GGTTCATATCACCTTACCGGCGCTTATCGCAGATTAGC
81	ACGCCCTTCATCGCCTCTGACTGCCAGG
82	GCATCCACCGTGTACGCTTAGTCGCTTA

Table S14

Table S14. Complementary *A. baumannii* 16S rRNA oligonucleotide sequences for ID `0331`.

Oligonucleotide Number	Sequence (5′→3′)
1	TAACTGAAGAGTTTGATCATGGCTCAGATTGAACGCTG
2	GCGGCAGGCTTAACACATGCAAGTCGAGCGGGGGAAGG
3	TAGCTTGCTACTGGACCTAGCGGCGGACGGGTGAGTAA
4	TGCTTAGGAATCTGCCTATTAGTGGGGGACAACATCTC
5	GAAAGGGATGCTAATACCGCATACGTCCTACGGGAGAA
6	AGCAGGGGATCTTCGGACCTTGCGCTAATAGATGAGCC
7	TAAGTCGGATTAGCTAGTTGGTGGGGTAAAGGCCTACC
8	AAGGCGACGATCTGTAGCGGGTCTGAGAGGATGATCCG
9	CCACACTGGGACTGAGACACGGCCCAGACTCCTACGGG
10	AGGCAGCAGTGGGGAATATTGGACAATGGGGGGAACCC
11	TGATCCAGCCATGCCGCGTGTGTGAAGAAGGCCTTATG
12	GTTGTAAAGCACTTTAAGCGAGGAGGAGGCTACTTTAG
13	TTAATACCTAGAGATAGTGGACGTTACTCGCAGAATAA
14	GCACCGGCTAACTCTGTGCCAGCAGCCGCGGTAATACA
15	GAGGGTGCGAGCGTTAATCGGATTTACTGGGCGTAAAG
16	CGTGCGTAGGCGGCTTATTAAGTC
17	GGATGTGAAATCCCCGAGCTTAACTtttggatatcactcattagtggt
18	TGGGAATTGCATTCGATACTGGTGAtttggatatcactcattagtggt
19	GCTAGAGTATGGGAGAGGATGGTAGtttggatatcactcattagtggt
20	AATTCCAGGTGTAGCGGTGAAATGCGTAGAGATCTGGA
21	GGAATACCGATGGCGAAGGCAGCCATCTGGCCTAATAC
22	TGACGCTGAGGTACGAAAGCATGGGGAGCAAACAGGAT
23	TAGATACCCTGGTAGTCCATGCCGTAAACGATGTCTAC
24	TAGCCGTTGGGGCCTTTGAGGCT
25	TTAGTGGCGCAGCTAACGCGATAAGtttggatatcactcattagtggt

26	TAGACCGCCTGGGGAGTACGGTCGCtttggatatcactcattagtggt
27	AAGACTAAAACTCAAATGAATTGACtttggatatcactcattagtggt
28	GGGGGCCCGCACAAGCGGTGGAGCATGTGGTTTAATTC
29	GATGCAACGCGAAGAACCTTACCTGGCCTTGACATACT
30	AGAAACTTTCCAGAGATGGATTGGTGCCTTCGGGAATC
31	TAGATACAGGTGCTGCATGGCTGTCGTCAGCTCGTGTC
32	GTGAGATGTTGGGTTAAGTCCCG
33	CAACGAGCGCAACCCTTTTCCTTACtttggatatcactcattagtggt
34	TTGCCAGCATTTCGGATGGGAACTTTAAGGATACTGCC
35	AGTGACAAACTGGAGGAAGGCGGGGACGACGTCAAGTC
36	ATCATGGCCCTTACGGCCAGGGCTACACACGTGCTACA
37	ATGGTCGGTACAAAGGGTTGCTACACAGCGATGTGATG
38	CTAATCTCAAAAAGCCGATCGTAGTCCGGATTGGAGTC
39	TGCAACTCGACTCCATGAAGTCGGAATCGCTAGTAATC
40	GCGGATCAGAATGCCGCGGTGAATACGTTCCCGGGCCT
41	TGTACACACCGCCCGTCACACCATGGGAGTTTGTTGCA
42	CCAGAAGTAGCTAGCCTAACTGCAAAGAGGGCGGTTAC
43	CACGGTGTGGCCGATGACTGGGGTGAAGTCGTAACAAG
44	GTAGCCGTAGGGGAACCTGCGGCTGGATCACCTCCTTA

Table S15

Table S15. Complementary *Salmonella* serovar 23S rRNA oligonucleotide sequences for IDs. The same set was used for all three serovar 13S rRNA ID assembly.

Oligonucleotide Number	Sequence (5′→3′)
1	CTTCGGCGTTGTAAGGTTAAGCCTCACGGTTCATTAGT
2	ACCGGTTAGCTCAACGCATCGCTGCGCTTACACACCCG
3	GCCTATCAACGTCGTCGTCTTCAACGTTCCTTCAGGAG
4	ACTCTAAGTCTCAGGGAGAACTCATCTCGGGGCAAGTT
5	TCGTGCTTAGATGCTTTCAGCACTTATCTCTTCCGCAT
6	TTAGCTACCGGGCAGTGCCATTGGCATGACAACCCGAA
7	CACCAGTGATGCGTCCACTCCGGTCCTCTCGTACTAGG
8	AGCAGCCCCCCTCAGTTCTCCAGCGCCCACGGCAGATA
9	GGGACCGAACTGTCTCACGACGTTCTAAACCCAGCTCG
10	CGTACCACTTTAAATGGCGAACAGCCATACCCTTGGGA
11	CCTACTTCAGCCCCAGGATGTGATGAGCCGACATCGAG
12	GTGCCAAACACCGCCGTCGATATGAACTCTTGGGCGGT
13	ATCAGCCTGTTATCCCCGGAGTACCTTTTATCCGTTGA
14	GCGATGGCCCTTCCATTCAGAACCACCGGATCACTATG
15	ACCTGCTTTCGCACCTGCTCGCGCCGTCA
16	CGCTCGCAGTCAAGCTGGCTtttggatatcactcattagtggt
17	TATGCCATTGCACTAACCTCtttggatatcactcattagtggt
18	CTGATGTCCGACCAGGATTAtttggatatcactcattagtggt
19	GCCAACCTTCGTGCTCCTCCGTTACTCTTTAGGAGGAG
20	ACCGCCCCAGTCAAACTACCCACCAGACACTGTCCGCA
21	ACCCGGGTAACGGGTCCACGTTAGAACATCAAACATTA
22	AAGGGTGGTATTTCAAGGTCGGCTCCATGCAGACTGGC
23	GTCCACACTTCAAAGCCTCCCACCTATCCTACACATCA
24	AGGCTCAATGTTCAGTGTCAAGCTATAGTAAAGGTTCA
25	CGGGGTCTTTCCGTCTTGCCGCGGTACACTGCATCTT

26	CACAGCGAGTTCAATTTCACTGAGTCTCGGGTGGAGAC
27	AGCCTGGCCATCATTACGCCATTCGTGCAGGTCGGAAC
28	TTACCCGACAAGGAATTTCGCTACCTTAGGACCGTTAT
29	AGTTACGGCCGCCGTTTACCGGGGCTTCGATCAGGAGC
30	TTCGCTTGCGCTGACCCCATCAATTAACCT
31	TCCGGCACCGGGCAGGCGTCtttggatatcactcattagtggt
32	ACACCGTATACGTCCACTTTCGTGTTTGCACAGTGCTG
33	TGTTTTTAATAAACAGTTGCAGCCAGCTGGTATCTTCG
34	ACTGACTTCAGCTCCATGAGTAAATCACTTCACCTACG
35	TGTCAGCGTGCCTTCTCCCGAAGTTACGGCACCATTTT
36	GCCTAGTTCCTTCACCCGAGTTCTCTCAAGCGCCTTGG
37	TATTCTCTACCTGACCACCTGTGTCGGTTTGGGGTACG
38	ATTTGATGTTACCTGATGCTTAGAGGCTTTTCCTGGAA
39	GCAGGGCATTTGTTGCTTCAGCAC
40	CGTAGTGCCTCGTCGTCACGCCTC
41	AGTGTTAAAGTGAACCGGATTTACCTGGAACACACACC
42	TACACGCTTAAACCGGGACAACCGTCGCCCGGCCAACA
43	TAGCCTTCTCCGTCCCCCCTTCGCAGTAACACCAAGTA
44	CGGGAATATTAACCCGTTTCCCATCGACTACGCCTTTC
45	GGCCTCGCCTTAGGGGTCGACTCACCCTGCCCCGATTA
46	ACGTTGGACAGGAACCCTTGGTCTTCCGGCGAGCGGGC
47	TTTTCACCCGCTTTATCGTTACTTATGTCAGCATTCGC
48	ACTTCTGATACCTCCAGCATGCCTCACGACACACC
49	TTCACAGGCTTACAGAACGCTCCCCTACCCAACAACAC
50	ACAGTGTCGCTGCCGCAGCTTCGGTGCATGGTTTAGCC
51	CCGTTACATCTTCCGCGCAGGCCGACTCGACCAGTGAG
52	CTATTACGCTTTCTTTAAATGATGGCTGCTTCTAAGCC
53	AACATCCTGGCTGTCTGGGCCTTCCCACATCGTTTCC
54	CACTTAACCATGACTTTGGGtttggatatcactcattagtggt

55	ACCTTAGCTGGCGGTCTGGGtttggatatcactcattagtggt
56	TTGTTTCCCTCTTCACGACGGACGTTAGCACCCGCCGT
57	GTGTCTCCCGTGATAACATTCTCCGGTATTCGCAGTTT
58	GCATCGGGTTGGTAAGCCGGGATGGCCCCCTAGCCGAA
59	ACAGTGCTCTACCCCCGGAGATGAATTCACGAGGCGCT
60	ACCTAAATAGCTTTCGGGGAGAACCAGCTATCTCCCGG
61	TTTGATTGGCCTTTCACCCCCAGCCACAGGTCATCCGC
62	TAATTTTTCAACATTAGTCGGT
63	TCGGTCCTCCAGTTAGTGTTtttggatatcactcattagtggt
64	ACCCAACCTTCAACCTGCCCtttggatatcactcattagtggt
65	ATGGCTAGATCACCGGGTTTCGGGT
66	CTATACCCTGCAACTTAACGCCCG
67	GTTAAGACTCGGTTTCCCTC
68	CGGCTCCCCTATTCGGTTAA
69	CCTTGCTACAGAATATAAGT
70	CGCTGACCCATTATACAAAA
71	GGTACGCAGTCACACCCAAA
72	GGGTGCTCCCACTGCTTGTACGTACACGGTTTCAGGTT
73	CTTTTTCACTCCCCTCGCCGGGGTTCTTTTCGCCTTTC
74	CCTCACGGTACTGGTTCACTATCGGTCAGTCAGGAGTA
75	TTTAGCCTTGGAGGATGGTCCCCCCATATTCAGACAGG
76	ATACCACGTGTCCCGCCCTACTCATCGAGCTCACAGCA
77	CATGCGCTTTTGTGTACGGGG
78	CTGTCACCCTGTATCGCGCGtttggatatcactcattagtggt
79	CCTTTCCAGACGCTTCCACTtttggatatcactcattagtggt
80	AACACACATGCTGATTCAGGtttggatatcactcattagtggt
81	CTCTGGGCTCCTCCCCGTTCGCTCGCCGCTACTGGGGG
82	AATCTCGGTTGATTTCTTTTCCTCGGGGTACTTAGATG
83	TTTCAGTTCCCCCGGTTCGCCTCATTAACCTATGGATT

84	CAGTTAATGATAGTGTGACGAATCACACTGGGTTTCCC
85	CATTCGGGTATCGCCGGTTATAACGGTTCATATCACCT
86	TACCGGCGCTTATCGCAGATTAGCACGCCCTTCATCGC
87	CTCTGACTGCCAGGGCATCCACCGTGT
88	ACGCTTAGTCGCTTAACCTCACAACCC

Table S16. Experimental details for nanopore measurements.

Figure	Sample	RNA ID	Buffer and pH	Voltage (mV)	Io open channel current (nA)	RNA ID frequency (events/min)	Mean translocation time for RNA IDs (ms)
Fig.1	*E.coli*	1311	4 M LiCl, pH 9.4	600	7.71	13	0.21
		1311	4 M LiCl, pH 9.4	600	6.60	20	0.22
		1311	4 M LiCl, pH 9.4	600	8.99	18	0.20
		1311	4 M LiCl, pH 9.4	600	9.06	22	0.19
	S. Typhimurium	3113	4 M LiCl, pH 9.4	600	9.87	14	0.25
		3113	4 M LiCl, pH 9.4	600	10.42	11	0.21
		3113	4 M LiCl, pH 9.4	600	13.41	16	0.26
Fig.2	*A. baumannii*	331, 3113	4 M LiCl, pH 9.4	600	7.95	2	0.24
		3113	4 M LiCl, pH 9.4	600	8.50	2	0.19
Fig.3	*S*. Newport	32213	4 M LiCl, pH 9.4	600	12.94	9	0.31
		32213	4 M LiCl, pH 9.4	600	9.93	9	0.34
		32213	4 M LiCl, pH 9.4	600	8.50	17	0.34
	S. Hadar	32213	4 M LiCl, pH 9.4	600	8.69	5	0.29
		32213	4 M LiCl, pH 9.4	600	12.09	14	0.27
	S. Typhimurium	32213	4 M LiCl, pH 9.4	600	6.56	13	0.11
		32213	4 M LiCl, pH 9.4	600	6.38	18	0.13
		32213	4 M LiCl, pH 9.4	600	8.72	4	0.14
Fig.4	*S*. Newport	220	2 M LiCl, pH 8.0	500	8.67	3	0.35
		220	2 M LiCl, pH 8.0	500	7.91	1	0.32
	S. Newport dCas9	22C	2 M LiCl, pH 8.0	500	9.10	2	0.32
			2 M LiCl, pH 8.0	500	9.11	7	0.27
	S. Enteritidis	220	2 M LiCl, pH 8.0	500	9.70	1	0.32
	S. Enteritidis dCas9	22C	2 M LiCl, pH 8.0	500	7.53	2	0.39
		22C	2 M LiCl, pH 8.0	500	8.29	2	0.38
	S. Typhi	220	2 M LiCl, pH 8.0	500	7.64	1	0.39
		220	2 M LiCl, pH 8.0	500	6.24	1	0.33
		220	2 M LiCl, pH 8.0	500	6.24	1	0.40
	S. Typhi dCas9	22C	2 M LiCl, pH 8.0	500	6.36	6	0.34

References

(1) Sandler, S. E.; Weckman, N. E.; Yorke, S.; Das, A.; Chen, K.; Gutierrez, R.; Keyser, U. F. Sensing the DNA-Mismatch Tolerance of Catalytically Inactive Cas9 via Barcoded DNA Nanostructures in Solid-State Nanopores. *Nat. Biomed. Eng.* **2023**, *8* (3), 325–334. https://doi.org/10.1038/s41551-023-01099-x.
